# Environmental health literacy among riverside residents in the Amazon region of Pará

**DOI:** 10.1590/1980-220X-REEUSP-2025-0015en

**Published:** 2025-10-20

**Authors:** Élida Fernanda Rêgo de Andrade, Sandy Isabelly Osório de Sousa, Sarah Souza de Carvalho, Débora de Cássia Quaresma Silva, Erlon Gabriel Rego de Andrade, Ana Kedma Correa Pinheiro, Ivaneide Leal Ataíde Rodrigues, Paula Gisely Costa Silva, Laura Maria Vidal Nogueira

**Affiliations:** 1 Universidade do Estado do Pará, Centro de Ciências Biológicas e da Saúde, Escola de Enfermagem Magalhães Barata, Belém, PA, Brazil.; 2 Universidade do Estado do Pará, Escola de Enfermagem Magalhães Barata, Programa de Pós-Graduação em Enfermagem, Belém, PA, Brazil.; 3 Universidade Federal do Rio de Janeiro, Escola de Enfermagem Anna Nery, Programa de Pós-Graduação em Enfermagem, Rio de Janeiro, RJ, Brazil.

**Keywords:** Health Literacy, Environmental Health, Rural Population, Rural Health, Public Health

## Abstract

**Objective:**

To analyze environmental health literacy and its impact on the daily lives of riverside residents in the Amazon region of Pará.

**Method:**

A descriptive, qualitative study conducted with 29 riverside residents at the Cotijuba Island Municipal Health Unit, in Belém, Pará, Brazil. Data were collected between April and July 2024 through semi-structured individual interviews. The *corpus* was subjected to lexical analysis using the *Interface de R pour les Analyses Multidimensionnelles de Textes et de Questionnaires* 0.7/alpha 2 software, using Descending Hierarchical Classification.

**Results:**

A total of 552 text segments were identified, of which 488 (88.41%) were used, generating seven lexical classes, organized into three thematic axes, which pointed out knowledge for dealing with the environment, the relationship between the environment and health, and the leading role and socio-environmental responsibility of riverside residents.

**Conclusion:**

Environmental health literacy among riverside residents involves the meanings they attribute to the environment that surrounds them, the adoption of care practices, and access to information on the topic. This is essential for them to understand how their actions impact individual and collective health.

## INTRODUCTION

Environmental health values the correlation between factors of the natural environment and human health, conditioned by anthropogenic modifications in the environment, as they influence and determine human beings’ quality of life^(1)^. In this regard, environmental conditions are fundamental determinants of health, while inequalities in access to information and resources interfere with the implementation of preventive measures and timely interventions^(2)^.

In view of this, communities living in rural, isolated and/or remote areas, have difficulty accessing basic services due to their biological, social, economic, intellectual and political particularities, as is the case of riverside populations, who actually resist and survive many human rights violations^(3)^. This group is characterized by a specific way of life, intrinsically associated with nature and based on the inheritance of sociocultural knowledge that shapes the development of daily activities, interpersonal relationships and healthcare practices^(4)^.

In the Brazilian Amazon, riverside communities emerged from historical processes of racial mixing and interaction between distinct peoples, making them vulnerable to social inequities. Despite fundamental citizenship rights, established in the 1988 Federal Constitution^(5)^, and health promotion actions outlined in the Brazilian National Policy for Comprehensive Health of Rural, Forest, and Water Populations (In Portuguese, *Política Nacional de Saúde Integral das Populações do Campo, da Floresta e das Águas* – PNSIPCFA), published in 2011^(6)^, the current situation for riverside communities is challenging and leads to social destitution. This reality impacts knowledge and the ability to form opinions on various topics, such as appropriate health interventions, disease prevention, and health promotion, involving the ability to make decisions aimed at maintaining or improving health and quality of life^(4,7)^. Thus, the concept of health literacy (HL) emerges, which encompasses the influence of social determinants and practical learning^(7)^.

HL promotes individuals’ autonomy to make informed choices^(7)^. According to the World Health Organization (WHO), HL encompasses individual knowledge, skills, and abilities, in accordance with organizational structures and available resources, which enable people to understand, assess, and use information and services, and have access to them, aiming to undertake or maintain quality health conditions and individual and collective well-being^(8)^.

From this perspective, environmental health literacy (EHL) consists of one of the spheres of HL, which associates elements and approaches from health and environmental and social sciences, contributing to a better understanding of issues related to exposure to risks and environmental degradation, in addition to the experiences of individuals with the environment in which they reside. It also aims to capture the diversity of skills and abilities that people need to seek, understand, assess and use environmental health knowledge to make informed decisions, with the purpose of protecting the environment and reducing risks that may, in the medium or long term, represent harm to individuals’ and groups’ health, configuring an emerging topic of study^(9)^.

Disparities in access to health, education, and information and communication technologies between riverside communities and the general population have an impact on the level of EHL, since the availability, dissemination, stimulation, and effectiveness of teaching/education and health practices influence how individuals behave in their relationship with the environment^(4,10)^.

EHL is considered to be both an educational process and a public health tool, with human health being closely related to the environment^(4,9)^. In this regard, it is necessary to understand riverside residents’ skills and abilities regarding the relationship between health and the environment in the local-regional context, aiming to support socio-environmental protection interventions and valuing biopsychosocial needs^(9)^.

Given the relevance of the topic and the need to share it, riverside residents’ EHL in the Amazon region of Pará was declared the object of this study. To develop this study, the following guiding questions were formulated: how does EHL manifest itself among riverside residents in the Amazon region of Pará? What factors can influence this literacy? How can riverside residents’ EHL affect their health? And how can EHL interfere with the relationships of riverside residents with the environment and the people they live with? Thus, the objective was to analyze environmental health literacy and its impact on the daily lives of riverside residents in the Amazon region of Pará.

## METHOD

### Study Design

A descriptive and qualitative study was chosen, using EHL as a conceptual basis, with the aim of characterizing, particularizing, and understanding various elements, such as social agents, behaviors, beliefs, decisions, and opinions that circulate in human relationships, as made possible by the qualitative approach^(11)^. Its writing was guided by the COnsolidated criteria for REporting Qualitative research (COREQ) guidelines, valuing three domains, inherent to the team, study design, and the set of analysis and results^(12)^.

It is worth noting that the quantitative approach has been widely incorporated into HL studies, with the use of validated instruments to measure levels of understanding and decision-making. However, since the stated objective of this study is to contemplate a traditional population’s EHL, a qualitative approach was chosen, given that this phenomenon requires a broader understanding of the context, associated with traditional knowledge, daily practices, forms of communication, access to information, and relationships with the environment in which participants are inserted. These aspects occur in a complex, particular, and subjective manner, permeated by social determinants of health and, therefore, could not be effectively accessed and captured with standardized scales and measures.

Thus, it is understood that the qualitative approach finds theoretical and methodological support, given the need to understand the meanings attributed to environmental health and its potential repercussions on subjects’ daily lives, generating evidence that strengthens knowledge about the reality investigated.

### Place

It took place at the Municipal Health Unit (MHU) of Cotijuba Island, located in the Administrative District of Outeiro (In Portuguese, *Distrito Administrativo de Outeiro* – DAOUT), municipality of Belém, state of Pará, Brazil. Managed by the Belém Municipal Health Department (In Portuguese, *Secretaria Municipal de Saúde de Belém* – SESMA), it offers Primary Health Care (PHC) services, associated with emergency, to meet the population’s demands.

With around 10,000 inhabitants, the island has undergone profound urban transformations in the last decade, due to its proximity to other districts of Belém, tourism activities and the inclusion of more inhabitants in the local dynamics, despite the weaknesses in the provision of public policies directed at some sectors, such as basic sanitation, solid waste, health surveillance, and public lighting^(13,14)^, characteristics that contributed to the choice of this setting.

Furthermore, the MHU constitutes the main link between the health system and the local population, fostering the consolidation of territorial ties within the community. It is a strategic setting within PHC, providing greater proximity to residents’ daily lives and providing opportunities for health promotion/recovery and disease prevention, fostering contact with people seeking care. Thus, it enabled qualified listening between users and the research team, a key element in investigating aspects related to EHL.

### Population and Selection Criteria

Twenty-nine riverside residents of both sexes, aged 18 or older, permanently residing on the island, regardless of whether they were enrolled in the MHU, participated. Only one riverside resident was excluded due to self-reported cognitive impairment, which limited his communication skills. There were no refusals or withdrawals. Therefore, the term “cognitive impairment” was used to pre-establish an exclusion criterion during the project development process, aiming not to compromise the content of the interviews.

Participants were selected by convenience, constituting the saturation sample, identified when the data obtained were sufficient to grasp the object of study, without requiring further interviews. Furthermore, the number of participants included met the literature recommendation, which indicates 20 to 30 participants as a satisfactory range for achieving objectives in qualitative studies^(15)^.

Therefore, data saturation was not considered as a numerical element, but as an analytical and reflective process, which values content analysis simultaneously with data collection, when the interviews begin to present recurrence of explanations and meanings, without adding relevant contributions to the object of study, capable of expanding or modifying the understanding of the phenomenon investigated^(15)^.

### Data Collection

To avoid potential bias, data were collected by four researchers who were properly supervised and aligned to express a similar approach during the interviews. To ensure data collection rigor, the research team was trained and the fieldwork was supervised by two professors. The researchers’ prior alignment aimed to minimize interference that could compromise the reports’ quality and authenticity and avoid eliminating the subjective dimension inherent in the data collection process. They were guided to use accessible, clear, and objective language, so that participants could understand the questions posed. This alignment was not intended to imply technical inflexibility, but rather to ensure due methodological rigor, guaranteeing respect for the narratives and exploring the aspects necessary to sufficiently understand the subject.

Approaching the setting, the researchers visited the MHU and introduced themselves to the professionals working there, discussing the research objectives and procedures. They learned about the service routine and scheduled days and times with the local manager for data collection, which took place in a private room on the unit’s premises. Therefore, professionals and participants had not met the researchers beforehand, nor were they aware of their aspirations and academic backgrounds, nor of the fact that this study was a requirement to complete the activities of the Institutional Scientific Initiation Scholarship Program (In Portuguese, *Programa Institucional de Bolsas de Iniciação Científica* – PIBIC). Therefore, the researchers dedicated the necessary time to familiarize themselves with the setting.

Riverside residents were approached individually in the waiting room, before or after their appointments, and informed about the research topic and invited to participate. To ensure comfort and privacy, those who agreed were led to a room where only the participant and a researcher remained. Details were presented and questions clarified in accessible language, obtaining formal acceptance.

The interviews took place from April to July 2024, individually, immediately after acceptance, without requiring an appointment, and were guided by a semi-structured script developed by the research team, covering two axes. With nine objective questions, the first axis explored participants’ sociodemographic profile through variables such as sex, age, race/skin color, religion, education level, professional activity, monthly family income, marital status, and cohabitation. With five subjective questions, developed through dialogue, the second axis explored their knowledge about environmental health, where and how they obtained it, whether they considered the environment can influence their life/health and how this might occur, what precautions they considered important for conserving the environment, and whether they applied this knowledge to prevent diseases/injuries and how they did so.

Given the data robustness, no need for repeat interviews was identified, nor were field diaries or additional collection techniques used. Although no pilot testing was conducted, the research team’s diligence in submitting the script for prior review by three nursing researchers with qualified scientific expertise in public health topics is noteworthy. These researchers suggested changes to the initial draft and endorsed the final version.

A copy was printed for each participant. Thus, responses to the questions in the first axis were recorded manually, while those in the second were documented in MPEG-1/2 audio layer 3 (MP3) format, using a digital recorder (Sony^®^ CD-PX240), with an average duration of 12 minutes. It is worth noting that the average duration of responses in the second axis was sufficient to achieve the objective and is similar to other studies that used qualitative analysis techniques with the same software used in this study, which were published in qualified scientific journals, reporting average durations of eight, nine, ten, and 18 minutes^(16, 17, 18, 19)^.

Furthermore, considering the sociocultural context, length was not used as the primary criterion for assessing the quality of reports, as in some cases, participants had limited access to schooling, which may have influenced their verbal expression, even with efforts to elicit larger volumes of statements through dialogue with the researchers. Despite the brevity of responses, the expression of subjectivity was clear, contributing to the completeness of results, as evidenced in the corresponding section.

### Data Analysis and Treatment

In Microsoft Office Excel^®^, version 2024, the sociodemographic profile data were organized into a spreadsheet and analyzed using descriptive statistics, generating absolute and relative values. The recordings were transcribed verbatim to form a *corpus* and subjected to lexical analysis in the *Interface de R pour les Analyses Multidimensionnelles de Textes et de Questionnaires* (IRaMuTeQ^®^), version 0.7, alpha 2 software.

Created by Pierre Ratinaud, IRaMuTeQ^®^ allows for a careful analysis of textual data by organizing it into a single, unformatted file (.txt) and separating it into text segments (TSs), highlighting frequent and representative words, statistical values, and the grammatical classification of words. It includes five analytical modalities: classical textual statistics; specificities and correspondence factor analysis; Descending Hierarchical Classification (DHC) or Reinert’s method, with a minimum satisfactory pass rate of 75%; similarity analysis; and word cloud analysis^(20,21)^.

Among the modalities, DHC was used, which detailed the TSs *corpus*, creating lexical classes according to the interdependence of the TSs that constituted them. Thus, the classes expressed particular meanings, learned from the reading of TSs, and were illustrated by a dendrogram, a visual representation that highlights the set of words that semantically characterize them^(20,21)^.

IRaMuTeQ^®^ assigned statistical values to these words, among which the frequency (F) of TSs, which present them in the classes, and the chi-square (X^2^), which indicates the associative strength of the words, are representative of those that obtained p < 0.0001^(20,21)^. It was decided to organize the classes into thematic axes, which were interpreted and discussed in light of EHL and the pertinent and updated scientific literature.

### Ethical Aspects

The ethical precepts of Resolutions 466/2012 and 580/2018 of the Brazilian National Health Council and the Ministry of Health, which respectively regulate research involving human beings and its ethical specificities within the Brazilian Health System (In Portuguese, *Sistema Único de Saúde*), were followed. Thus, the study was authorized by SESMA and approved by the Research Ethics Committee of the undergraduate nursing program at the *Universidade do Estado do Pará*, under Opinion 6,637,778, issued in February 2024. Participants read and signed the Informed Consent Form, and their identities were guaranteed confidentiality with alphanumeric codes consisting of the letter R, for “riverside resident”, followed by an Arabic numeral indicating the order of the interviews.

## RESULTS

Among participants, females predominated (n = 24; 82.76%), and their ages ranged from 20 to 86 years, with emphasis on the age groups 20 to 24 (n = 5; 17.24%) and 45 to 49 years (n = 5; 17.24%), with an average of 46.55. Regarding race/skin color, the largest proportion was brown (n = 22; 75.86%), and regarding religion, there was a predominance of evangelicals (n = 18; 62.07%). The most significant levels of education were complete high school (n = 11; 37.93%) and incomplete elementary school (n = 11; 37.93%). The majority (n = 16; 55.17%) declared having some paid activity, having a monthly family income of up to one minimum wage (n = 19; 65.52%), being married or living in a consensual union (n = 17; 58.62%) and living with one or more people (n = 27; 93.10%), the majority of whom were their children (n = 28; 96.55%).

**Figure 1:**
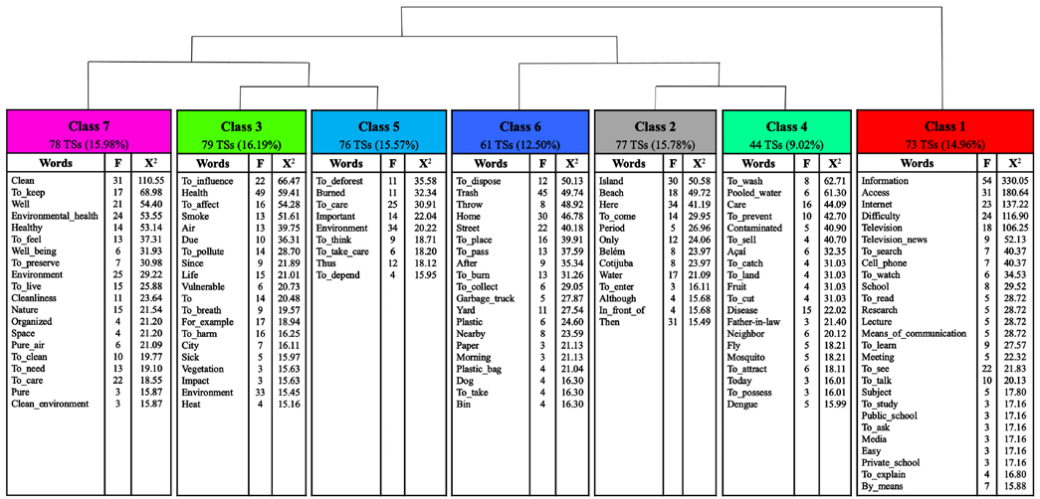
Descending Hierarchical Classification dendrogram. Belém, Pará, Brazil, 2024.

In IRaMuTeQ^®^, the *corpus* consists of 29 texts, corresponding to the total number of interviews, generating 552 TSs, of which 488 (88.41%) were used. A total of 19,165 occurrences (forms or words) were identified, with 2,454 distinct words and 1,300 hapaxes, i.e., words with a frequency of one, representing 6.78% of occurrences and 52.97% of distinct words. The average number of words per TS was approximately 34.72. Two *subcorpora* with seven lexical classes were structured, as illustrated in the DHC dendrogram (Figure 1). Following the partition order, class 1 comprised the first *subcorpus*, and classes 6, 7, 2, 4, 3, and 5 comprised the second so that classes 6 and 7 were generated simultaneously as well as the pair 2 and 4 and the pair 3 and 5.

These classes were organized into three thematic axes. The first axis consisted of classes 7, 6, and 5, titled “Meanings attributed to environmental health”, “Behaviors toward the environment”, and “Practices to preserve the environment”. Composing the second axis, classes 3 and 2 were titled “Influences of the environment on health” and “Perceptions of the local context”. In turn, classes 4 and 1 comprised the third axis, titled “Practices to prevent diseases and injuries” and “Challenges and ways to access information about health and the environment”. These axes were named according to the content of their classes and are presented below, with some emblematic excerpts.

### Thematic Axis 1 – Knowledge that Guides Ways of Dealing with the Environment (Classes 7, 6 and 5)

Comprising 78 TSs (15.98% of the *corpus*) and 20 representative words (p < 0.0001), class 7 encompasses ways of understanding environmental health based on knowledge about it, experienced processes, and the reality in which the participants lived. In this sense, words such as “clean” (X^2^ = 110.55), “to keep” (X^2^ = 68.98), and “well” (X^2^ = 54.40) highlight relationships between the notion of cleanliness and the imagery of a healthy environment conducive to the well-being of the individuals who interact within it:

*Environmental health, for me, is the environment where I live. It’s necessary to take care of garbage collection and a little bit of everything, because I believe the environment needs to be clean and organized.* (R9)

*The environment can influence my health and well-being throughout my life. Therefore, I believe we must maintain cleanliness to have a clean environment.* (R25)

The interconnection between environmental health, quality of life and happiness of participants was observed, linking physical and mental well-being to living in pollution-free environments with preserved nature:

*Environmental health is living well in a peaceful, pollution-free place.* (R21)

*If you don’t breathe clean air, you won’t be well. Therefore, nature is an environment that needs to be always pure, clean and healthy, for you to feel happy.* (R24)

It was noted that participants’ knowledge of the concept of environmental health was incipient, as they related it to the visible aspects of the environment, meaning they did not explain many of the elements that constitute it, such as the ecological interaction between living beings, the aerial and climatic conditions of nature and the social relationships built in this context:

*The* [lack of] *environmental health, in my view, is having too much trash in the water and soil.* (R4)

*Environmental health is an environment without trash on the streets, preserving nature and rivers.* (R15)

Composed of 61 TSs (12.50% of the *corpus*) and 20 representative words (p < 0.0001), class 6 elucidates the participants’ behaviors regarding the environment, constructing the idea that they acted according to the possibilities of the reality they experienced. The word “trash” (X^2^ = 49.74), which presented the second highest chi-square of the class, associated with the verb “to dispose” (X^2^ = 50.13) and the word “throw” (X^2^ = 48.92), indicates inadequate practices of separation, storage and disposal of solid waste:

*When it comes to trash,* [at home] *there’s no separation, and all kinds of trash are thrown into the street.* (R12)

*I throw trash I can’t burn out into the street, so it doesn’t pile up and end up in the yard.* (R15)

The lack of an adequate waste collection system favored the adoption of alternatives to solve the accumulation of domestic waste, which could generate negative repercussions for health and the environment:

*We burn the trash to avoid it piling up and attracting insects.* (R10)

*I burn the trash because sometimes the garbage truck comes by and doesn’t pick up all the trash from the street. To avoid putting it in front of my house and attracting mosquitoes, I prefer to burn it.* (R24)

Composed of 76 TSs (15.57% of the *corpus*) and nine representative words (p < 0.0001), class 5 expresses everyday practices for environmental conservation. In this sense, the words “to deforest” (X^2^ = 35.58), “burned” (X^2^ = 32.34), and “to care” (X^2^ = 30.91) simultaneously refer to the concern and need to preserve the environment, including the protection of forests and rivers. Furthermore, they refer to the notion that avoiding environmentally degrading practices helps improve nature:

*It’s important not to deforest the environment. When I talk about the environment, I mean the forest, including the fish and everything in the rivers. It’s important not to dump trash and plastic bags.* (R1)

*I think everyone should think about caring for the environment, and therefore, people shouldn’t set fires, to help reduce air pollution and keep the environment clean.* (R19)

The repetition of the verb “to depend” (X^2^ = 15.95), in TSs of this class, gives meaning to the importance of environmental preservation, correlated with riverside populations’ sustenance and health in terms of food and income sources, configured especially by agriculture and fishing:

*If humans don’t care for the environment, it can cause disease. Therefore, we have to take care of the rivers so they don’t die, because we depend on them to catch shrimp and fish.* (R1)

*Where I lived, I was accustomed to taking good care of the trees and not cutting them down, because many fruits come from trees, especially mango and açaí trees, especially because we depend on them economically today.* (R3)

### Thematic Axis 2 – Perspectives on the Relationship between Environment and Health (Classes 3 and 2)

In its formation, class 3 presented 79 TSs (16.19% of the *corpus*), being the largest class, and 20 representative words (p < 0.0001), among which stand out “to influence” (X^2^ = 66.47), “health” (X^2^ = 59.41), “to affect” (X^2^ = 54.28) and “smoke” (X^2^ = 51.61). These words express the recognition of the relationship between the environment and health conditions, demonstrating the vulnerability of human health to environmental degradation and environmental pollution as an aspect that facilitates the transmission of diseases:

*I burn forests, and I think this makes my health more vulnerable to the environment, because the smoke from the fires is harmful to health.* (R12)

*I believe that pollution and deforestation have significant impacts on life and health. The air I breathe is affected by smoke from the fires and by fuels and factories that pollute a lot.* (R18)

It was possible to capture reports that elucidate participants’ understanding of the various factors contained in the environment that predispose them to illness, with the possibility of worsening this setting, through the spread of diseases in the community:

*A bad environment harms our health and can cause various diseases, such as rat disease, called leptospirosis, and many others.* (R3)

*There are things that can affect our health, such as stagnant water, dengue fever, garbage, and wildfires. Smoke is bad for me because I have chronic bronchitis, which I acquired because of it* [referring to the wildfires]. (R5)

Composed of 77 TSs (15.78% of the *corpus*) and 13 representative words (p < 0.0001), class 2 evidenced the riverside residents’ sense of belonging to the local context, through elements associated with their own residence and occupational activities, but also with the environmental pollution resulting from residents’ actions, added to the inappropriate disposal of waste by visitors, portraying the impacts of tourism on the environment. Words such as “island” (X^2^ = 50.58), “beach” (X^2^ = 49.72), and “water” (X^2^ = 21.09) denote this perspective:

*When the holidays arrive, things get much worse, because people arrive on the island, make a mess, and then leave, leaving the streets in disrepair. But the people who live here can’t leave because their houses are right here.* (R5)

*We work on the beach and live across the street, so we’re constantly collecting trash because people from outside, and even locals, use the area and leave trash behind.* (R12)

In this context, some statements revealed a feeling of abandonment by the public administration and a lack of basic sanitation on the island, which could compromise the health and quality of life of residents. This situation was aggravated by problems with garbage collection, which resulted in environmental contamination and the degradation of natural resources, corroborating the need for effective public policies to meet the population’s needs and preserve the environment:

*Here on the island, we have no sewage treatment or water treatment. The community is having problems with garbage collection, and as a result, the environment is contaminated.* (R12)

*We are severely neglected by public authorities, the government, and everyone who manages our island. Although there is a lot of tourism, the island is neglected by the government.* (R27)

### Thematic Axis 3 – Leading Role of Riverside Residents and Socio-Environmental Responsibility (Classes 4 and 1)

Composed of 44 TSs (9.02% of the *corpus*) and 20 representative words (p < 0.0001), class 4 is the smallest. It portrays the knowledge and learning acquired among riverside residents to maintain health and implement measures to protect them from possible diseases and injuries, as evidenced by the words “care” (X^2^ = 44.09), “to prevent” (X^2^ = 42.70), “contaminated” (X^2^ = 40.90), “to catch” (X^2^ = 31.03), and “disease” (X^2^ = 22.02). These measures constituted practices that emerged from prior, often incipient, information obtained and shared based on traditional experiences and knowledge:

*At home, I’m careful with trash, while others aren’t and leave it in the street. The leachate then goes into the groundwater, and we use contaminated well water. Therefore, I’m concerned about contracting diseases from this water.* (R10)

*To prevent illness, I stay away from people who may be infected.* (R16)

The words “to wash” (X^2^ = 62.71) and “pooled water” (X^2^ = 61.30) had the two highest chi-squared values in the class, reflecting participants’ concern for environmental hygiene and vector control to prevent microorganisms and insects from reproducing in hot, humid places, favored by the local climate. They also expressed concern about avoiding stagnant water to control dengue fever:

*I remove any falling açaí leaves to prevent pooling, I turn the bowls over to prevent water from accumulating, I’m careful with the water I store, and I cover everything. I think this is good because it prevents dengue fever and other diseases.* (R15)

*Açaí is proof that you can contract diseases. For example, if a barber bug lands on the açaí and you don’t wash it or take care of it, you can get sick. My father-in-law got sick because he wasn’t careful.* (R22)

Composed of 73 TSs (14.96% of the *corpus*) and 27 representative words (p < 0.0001), class 1 addresses some challenges and the means by which riverside residents accessed information related to health and the environment. Words such as “information” (X^2^ = 330.05), “access” (X^2^ = 180.64), and “difficulty” (X^2^ = 116.90) portray important gaps, experienced by some participants (n = 13; 44.83%), in accessing information on the topic throughout their lives. This is due to the lack of resources and infrastructure and the deficit in the quality of the content made available to this population, distancing them from reliable sources:

*Those without cell phones, television, and internet access cannot access information, and there are people in Cotijuba who don’t.* (R5)

*It’s very difficult to access health information through the media, and when it is available, the information is superficial.* (R12)

Among the means of access available to the other portion of participants (n = 22; 75.86%), the words “internet” (X^2^ = 137.22), “television” (X^2^ = 106.25), “television news” (X^2^ = 52.13), and “cell phone” (X^2^ = 40.37) indicate the digital sources used in daily life to obtain information. Thus, it was identified how much they helped disseminate knowledge and practices, influencing decision-making and promoting autonomy in the search for new knowledge. The importance attributed to healthcare professionals who, during home visits, contributed explanations on the topic was also noted:

*I also research online and on YouTube* ^®^ *. These days, it’s very advanced. There are many media outlets and a lot of good information about the environment.* (R9)

*I see a lot of information about it online, on my cell phone, and in the news broadcast on television, in addition to information provided by doctors and community health workers, who are always helping and explaining.* (R18)

The term “lecture” (X^2^ = 28.72) stood out, demonstrating the desire for more information, with appropriate guidance that can be incorporated into the local reality. This highlighted the need for initiatives that educate and raise awareness among residents:

*I certainly encounter several difficulties in accessing information. It would be great if we had a way to access it on the island, like a lecture, but we don’t*. (R13)

[Healthcare professionals] *could hand out a brochure with a time and place to explain it to us, because we don’t know about this information.* (R25)

## DISCUSSION

The results show that riverside residents’ perceptions of environmental health were related to elements of their daily lives and were influenced by the social determinants of health, which shape their lifestyles and the environmental conditions they experience^(22)^.

Riverside communities’ limited educational attainment can influence economic development and impact access to consumer goods, such as resources for mobility to geographically distant healthcare services, creating barriers to both access to and adequate understanding of health and environmental information^(23)^. This interferes with the ways in which this population manages their living and health conditions in an environment marked by territorial specificities, a situation that involves EHL^(10,24)^.

In the context of these reflections, the discussion was organized according to the logic of the results, structuring it through the sections presented below.

### Thematic Axis 1 – Knowledge that Guides Ways of Dealing with the Environment (Classes 7, 6 and 5)

It is known that the cultural context of riverside residents shapes conceptions and behaviors regarding interpersonal relationships and the environment in which they live, influencing direct coexistence with nature, permeated by biopsychosocial elements that characterize this group^(25)^. Thus, their interpretations revealed a practical understanding of environmental health, associating it with specific daily efforts to maintain the environment without inappropriate disposal of solid waste, supporting the construction of an environmental awareness resulting from previous experiences.

Understandings like this transcend the notion of dirt and pollution, by attributing a sense of well-being to those who transit and interact in a well-maintained space. This expresses a form of harmonious coexistence with the environment, as natural elements are conceived as components of cultural identity and ways of life. From this perspective, affections can be attributed to the environment and associated with the appreciation of local practices and knowledge^(25)^.

This demonstrates that a clean environment provides favorable conditions for health, highlighting the links between environmental practices and the prevention of risks and injuries, which favors the promotion of a healthy environment, aligned with quality of life. However, the limited knowledge that participants expressed when conceptualizing environmental health demonstrated a reductionist view, with an emphasis on solid waste and water bodies, as the reality they faced highlighted precarious sanitary conditions, coupled with the fact that they depended on natural resources.

This limitation can be understood by considering that EHL is associated with several factors, including sociodemographic characteristics, information sources, and daily practices^(10)^. In this context, unsatisfactory levels of education offered to rural populations ultimately hinder their ability to obtain reliable information on health-related aspects. Such disadvantages hinder good care management, disease prevention and informed decision-making^(3)^.

Furthermore, the poor infrastructure of basic sanitation services led riverside residents to adopt alternative measures to avoid the accumulation of waste in social gathering areas, which could harbor rodents and serve as breeding grounds for mosquitoes, especially in containers that accumulated water, increasing the risk of contracting dengue fever and other pathologies related to environmental degradation. To ensure health and safety at home, initiatives based on open-air waste burning have been reported. However, it is worth noting that the emission of pollutants into the atmosphere can reduce immunity and increase the occurrence of allergies, infections, and inflammatory processes in various anatomical and physiological structures, such as the nose and throat^(26)^.

### Thematic Axis 2 – Perspectives on the Relationship between Environment and Health (Classes 3 and 2)

Participants recognized the impacts of environmental degradation on human health, by associating deforestation, the emission of polluting gases and urbanization with the development of respiratory pathologies experienced by them^(27)^. This reality reveals unfavorable environmental conditions as factors that determine populations’ health, especially vulnerable communities that lack basic sanitation, including everything from the lack of infrastructure for sewage and drinking water distribution to the inadequate disposal of waste^(28)^.

In an attempt to clarify forms of illness resulting from environmental pollution, participants recalled previous conditions to point out factors that made them predisposed to illness, characterizing the poorly maintained environment as facilitating the occurrence and spread of infectious diseases^(23)^. In this process, subjects draw on their experiences to explain facts, striving to find answers to the source of problems, anchoring themselves mainly in common sense^(29)^.

Perceptions of the local context highlighted the representation of the environment as a space of sustenance and belonging, where the irresponsible actions of residents and visitors were linked to water, soil, and air pollution, generating environmental imbalances. In such settings, tourists are perceived as a new element, leaving their mark on the spaces already occupied by local actors^(30)^.

External actions characterized by environmental degradation tend to impact how residents assign meaning to and act toward the environment and the people they live with. It is recognized that the involvement of traditional populations in tourism is a growing phenomenon, but it is necessary to prioritize the landscape, cultural identity, and the preservation of natural resources, valuing the knowledge and cosmologies unique to each group^(30)^.

A feeling of neglect by public authorities was also identified, as they failed to effectively address basic sanitation needs, including waste collection. Historically, rural communities lack resources that could encourage improvements in quality of life, including access to education, healthcare, and sanitation^(23)^. The Millennium Development Goals Report, published in 2015, exposed the contrasts in living conditions between urban and rural areas, noting that access to drinking water and sanitation were four and three times lower in rural areas, respectively, compared to urban areas. Furthermore, children were four times more likely to be out of school in rural areas than in urban areas, impacting morbidity and mortality indicators^(31)^.

In this context, the Sustainable Development Goals express quality of life as a universal right, which must be guaranteed to everyone, and, to achieve this, each person needs opportunities to access health knowledge that is compatible with their level of understanding, making them capable of making appropriate decisions in their daily lives^(22)^.

### Thematic Axis 3 – Leading Role of Riverside Residents and Socio-Environmental Responsibility (Classes 4 and 1)

Riverside communities live with a history of climate change in their environment, particularly with the rising and falling of rivers. This reality culminates in popular knowledge that guides practices to address the region’s characteristic inequities^(32)^.

The behaviors reported by these individuals demonstrate recognition of basic hygiene and nature conservation measures to mitigate disease risks, emphasizing the EHL’s description of competencies and skills for understanding, assessing, and using environmental health information^(10,26)^. Adherence to health measures was justified by the need to conserve the environment, including fauna, flora, surface and groundwater, soil, and air. This concept can result in better health indicators due to greater protection against diseases and conditions related to degradation, as reiterated in the literature^(26)^.

Inequalities in access to reliable information weaken riverside residents’ learning process on this topic, limiting their ability to understand and implement interventions aimed at maintaining health and conserving the environment. Such disparities are generally influenced by a lack of financial and technological resources, poor infrastructure, a shortage of educational initiatives, and limited access to basic services, such as health and education^(33)^.

Despite the challenges they faced in obtaining information about health and the environment through their own initiatives, the growing use of the internet as a resource that allows access to diverse content and can reach a large portion of the population became evident, as pointed out in the literature^(7)^. Thus, digital media can bring geographically isolated groups closer to information on this and other topics, helping them seek practical solutions and strengthen their autonomy in decision-making^(32)^.

Another important aspect is home visits conducted by healthcare professionals, which provide a valuable opportunity for interaction between the team and the community, fostering the sharing of information, clarifying doubts, and promoting preventive practices, according to each individual’s biopsychosocial needs. During these visits, in addition to assessing living and health conditions, it is possible to assess environmental conditions in detail, enabling the identification of potential risks and the provision of contextualized guidance, especially for those who lack easy access to information or have difficulty understanding^(23)^.

A desire for more information was expressed, indicating that participants recognized the need to better understand how their actions affected not only their living conditions and health, but also public health and the quality of resources available in the environment. Therefore, it is essential to promote dialogical and emancipatory educational initiatives to empower the population and facilitate their engagement, raising awareness about creating collaborative networks with the goal of strengthening health and quality of life, in line with environmental preservation^(33,34)^.

The limitations of this study include the fact that it was developed in a specific setting, with particular economic, political, sociocultural, and territorial characteristics, restricting the geographic scope of the data. However, the results and the insights that emerged from them can contribute significantly to the fields of nursing and public health, by providing support for comparing and interpreting the results of other studies in similar contexts, improving care actions in riverside communities regarding environmental health, and developing public policies, educational programs, and other initiatives aimed at health promotion and environmental protection.

## CONCLUSION

It has been demonstrated that riverside residents’ EHL in the Amazon region of Pará involves attributing meaning to their surroundings, adopting practices to care for the environment and their health, and accessing information on the topic. Therefore, it is essential for riverside residents to understand how their actions impact individual and collective health, involving the interrelationship between hygiene practices, basic sanitation, and environmental protection. However, factors such as limited access to information, unfavorable socioeconomic conditions, and a lack of qualified educational initiatives can hinder EHL, interfering with the ability to make decisions based on environmental conditions.

The impact of EHL on riverside residents’ health can be significant, helping to prevent illness and promote a healthy lifestyle. From this perspective, raising awareness about the need to preserve the environment can strengthen riverside residents’ relationships with their surroundings, fostering a sense of community and establishing shared responsibility. Therefore, investing in education and improving the quality of the actions implemented is essential to empower these people and, thus, make them active agents in preserving the environment and improving their living and health conditions.

Based on the data, it is understood that it will be possible to generate further robust scientific knowledge through research activities in nursing and related fields to support or foster the creation, implementation, and effectiveness of public policies targeted to the needs of these populations. By being culturally and socially adapted, these policies can contribute to equity in access to healthcare and environmental preservation in historically vulnerable territories.

In this context, nursing professionals, especially nurses, can act to promote health, enabling individuals and groups to access, understand, assess, and utilize knowledge about health and the environment to make critically informed decisions, empowering themselves to care for themselves and their surroundings, and becoming agents of change in their communities. This highlights the role of nurses as educators, given the social context of riverside residents, marked by low educational levels and limited access to information.

## Data Availability

The data supporting this study are available upon request to the corresponding author.

## References

[1] Brasil (2022). Ministério da Saúde. Ministério da Educação. Caderno temático do Programa Saúde na Escola: saúde ambiental [Internet].

[2] Martins C, Alho AM, Muquinapir F, Madeira F, Durão J, Lima LF. (2024). Environmental determinants of health: NOVA National School of Public Health research to tackle ongoing threats and challenges. Port J Public Health.

[3] Santos IO, Rabello RED, Corrêa RG, Melo GZS, Monteiro AX. (2021). Avanços e desafios na saúde das populações ribeirinhas na região amazônica: uma revisão integrativa. Rev APS..

[4] Parmejiani EP, Queiroz ABA, Cunha MPL, Carvalho ALO, Santos GS, Bezerra JF. (2022). Riverside men’s knowledge and ways of acting regarding condom use. Texto Contexto Enferm.

[5] Brasil (2016). Senado Federal. Secretaria de Editoração e Publicações. Coordenação de Edições Técnicas. Constituição da República Federativa do Brasil: texto constitucional promulgado em 5 de outubro de 1988, com as alterações determinadas pelas Emendas Constitucionais de Revisão nº 1 a 6/94, pelas Emendas Constitucionais nº 1/92 a 91/2016 e pelo Decreto Legislativo nº 186/2008 [Internet].

[6] Brasil (2014). Ministério da Saúde Portaria nº 2.331, de 23 de outubro de 2014. Altera a Portaria nº 2.866/GM/MS, de 2 de dezembro de 2011, que institui, no âmbito do Sistema Único de Saúde (SUS), a Política Nacional de Saúde Integral das Populações do Campo e da Floresta (PNSIPCF). Diário Oficial da União [Internet].

[7] Pfleger E, Drexler H, Lutz R. (2024). Health literacy and environmental risks focusing air pollution: results from a cross-sectional study in Germany. Int J Environ Res Public Health.

[8] World Health Organization (2021). Health promotion glossary of terms 2021 [Internet].

[9] Lindsey M, Chen S-R, Ben R, Manoogian M, Spradlin J. (2021). Defining environmental health literacy. Int J Environ Res Public Health.

[10] Bert F, Gea M, Previti C, Massocco G, Lo Moro G, Scaioli G. (2023). The environmental health literacy of Italian general population: the SPeRA cross-sectional study. Int J Environ Res Public Health.

[11] Firmo JOA, Peixoto SV, Souza GA, Loyola AI. (2020). Evolution of publications on health of the older adults in the Journal Ciência & Saúde Coletiva. Ciênc Saúde Colet.

[12] Souza VRS, Marziale MHP, Silva GTR, Nascimento PL. (2021). Translation and validation into Brazilian Portuguese and assessment of the COREQ checklist. Acta Paul Enferm.

[13] Pereira NSS, Tavares MGC. (2020). A questão do turismo em Cotijuba, Belém - PA: mudanças, permanências e (co)existências no cotidiano ilhéu. Cad Virtual Tur.

[14] Silva EK, Almeida AS, Gama LHOM. (2021). Ilhas ameaçadas com o desflorestamento: análise da fragmentação florestal da ilha de Cotijuba, Belém, Pará, Brasil. Bol Mus Para Emílio Goeldi Ciênc Nat.

[15] Minayo MCS. (2017). Amostragem e saturação em pesquisa qualitativa: consensos e controvérsias. Rev Pesq Qual [Internet].

[16] Nonato LOF, Peres AM, Khalaf DK, Souza MAR, Figueiredo KC, Lapierre J. (2020). Primary Healthcare management strategies in socially vulnerable territories exposed to violence. Rev Esc Enferm USP.

[17] Miranda ALC, Sagica TP, Nicolussi AC, Parente AT, Toffano SEM, Ramos AMPC. (2024). Nursing professionals’ perceptions and challenges regarding difficult peripheral venipunctures in oncology. Rev Enferm UERJ..

[18] Santos IFSO, Rodrigues LSA, Suto CSS. (2024). Abortion from the perspective of undergraduate nursing students. Enferm (Montev).

[19] Oliveira JC, Borges F, Tonini NS, Maraschin MS, Bernardino E. (2023). Performance of hospital nurses in the management of the COVID-19 crisis. Rev Enferm UERJ..

[20] Góes FGB, Santos AST, Campos BL, Silva ACSS, Silva LF, França LCM. (2021). Use of IRAMUTEQ software in qualitative research: an experience report. Rev Enferm UFSM..

[21] Sousa YSO. (2021). O uso do software Iramuteq: fundamentos de lexicometria para pesquisas qualitativas. Estud Pesqui Psicol.

[22] Moyses RA, Toporcov TN, Cunha LAS., Costa FP, Castro AM, Leão N. (2024). Letramento em saúde: métrica para apoiar a educação escolar a se tornar base para uma vida saudável. organizadores. 1ª Mostra Brasileira de Literacia em Saúde: pistas para o SUS e as políticas públicas [Internet].

[23] Guimarães AF, Barbosa VLM, Silva MP, Portugal JKA, Reis MHS, Gama ASM. (2020). Access to health services for riverside residents in a municipality in Amazonas State, Brazil. Rev Pan-Amaz Saúde.

[24] Zanchetta MS, Moraes KL. (2023). Health literacy: a challenging social determinant of health for nursing research and practice. Rev Baiana Enferm..

[25] Santos ÉR, Leal RS, Veras ATR, Maia ROS. (2020). Geograficidade amazônica: percepção do lugar dos ribeirinhos de Sacaí, Baixo Rio Branco – RR. Acta Geogr.

[26] Abubakar IR, Maniruzzaman KM, Dano UL, AlShihri FS, AlShammari MS, Ahmed SMS. (2022). Environmental sustainability impacts of solid waste management practices in the global south. Int J Environ Res Public Health.

[27] Berman JD. (2024). Air pollution and health – new advances for an old public health problem. JAMA Netw Open.

[28] Batista LM, Neu V. (2024). Olhares para o sanear: as percepções de ribeirinhos sobre uma experiência com tecnologias sociais na Amazônia Oriental. Rev Bras Estud Urbanos Reg..

[29] Nóbrega DO, Andrade ERG. (2021). Teoria das Representações Sociais e racionalidades distintas: tensionamentos e sínteses entre a ciência e o senso comum. Comun Soc.

[30] Coelho EÁ, Gontijo BM. (2021). O processo de organização para o turismo nas comunidades ribeirinhas da Reserva Amanã, AM. Tur Soc.

[31] United Nations (2015). Millennium Development Goals Report 2015 [Internet].

[32] Gama ASM, Secoli SR. (2020). Self-medication practices in riverside communities in the Brazilian Amazon Rainforest. Rev Bras Enferm.

[33] Ferreira EI, Nascimento MHR. (2024). Educação ambiental como instrumento de empoderamento e garantia dos direitos humanos das comunidades tradicionais no estado do Amazonas. RevBEA – Rev Bras Educ Ambient.

[34] Silva MGO, Furtado RCS, Sena MLM, Naka KS, Souza JS, Castro NJC. (2024). Environmental education in teaching-service-community integration practices: application of educational technologies in the waiting room. Esc Anna Nery.

