# Supplementary material for: Environmental health literacy among riverside residents in the Amazon region of Pará
Source: Rev Esc Enferm USP. 2025 Oct 20;59:e20250015. doi: 10.1590/1980-220X-REEUSP-2025-0015en (PMC12543437; doi:10.1590/1980-220X-REEUSP-2025-0015en)
Supplement: Translation

# Translation: Letramento em saúde ambiental entre ribeirinhos da Amazônia paraense

**DOI:** 10.1590/1980-220X-REEUSP-2025-0015pt

**Authors:** Élida Fernanda Rêgo de Andrade, Sandy Isabelly Osório de Sousa, Sarah Souza de Carvalho, Débora de Cássia Quaresma Silva, Erlon Gabriel Rego de Andrade, Ana Kedma Correa Pinheiro, Ivaneide Leal Ataíde Rodrigues, Paula Gisely Costa Silva, Laura Maria Vidal Nogueira

**Affiliations:** 1 Universidade do Estado do Pará, Centro de Ciências Biológicas e da Saúde, Escola de Enfermagem Magalhães Barata, Belém, PA, Brasil.; 2 Universidade do Estado do Pará, Escola de Enfermagem Magalhães Barata, Programa de Pós-Graduação em Enfermagem, Belém, PA, Brasil.; 3 Universidade Federal do Rio de Janeiro, Escola de Enfermagem Anna Nery, Programa de Pós-Graduação em Enfermagem, Rio de Janeiro, RJ, Brasil.

**Keywords:** Letramento em Saúde, Saúde Ambiental, População Rural, Saúde da População Rural, Saúde Pública

## Abstract

**Objetivo:**

Analisar o letramento em saúde ambiental e as suas repercussões no cotidiano de ribeirinhos da Amazônia paraense.

**Método:**

Estudo descritivo e qualitativo, realizado com 29 ribeirinhos na Unidade Municipal de Saúde da Ilha de Cotijuba, em Belém, Pará, Brasil. Os dados foram coletados entre abril e julho de 2024, por meio de entrevistas individuais semiestruturadas, cujo *corpus* foi submetido à análise lexical no *software Interface de R pour les Analyses Multidimensionnelles de Textes et de Questionnaires* 0.7/*alpha* 2, utilizando a Classificação Hierárquica Descendente.

**Resultados:**

Identificaram-se 552 segmentos de texto, dos quais foram aproveitados 488 (88,41%), gerando sete classes lexicais, organizadas em três eixos temáticos, que apontaram saberes para lidar com o meio ambiente, relação entre meio ambiente e saúde, protagonismo e responsabilidade socioambiental dos ribeirinhos.

**Conclusão:**

O letramento em saúde ambiental de ribeirinhos envolve os significados que atribuem ao cenário que os cerca, a adoção de práticas de cuidado e o acesso a informações sobre o tema, sendo fundamental para que compreendam como suas ações repercutem na saúde individual e coletiva.

## INTRODUÇÃO

A saúde ambiental valoriza a correlação entre os fatores do meio natural e da saúde humana, condicionada às modificações antrópicas no ambiente, pois elas influenciam e determinam a qualidade de vida do ser humano^(1)^. Nesse sentido, as condições ambientais são determinantes fundamentais da saúde, enquanto que as desigualdades no acesso às informações e aos recursos interferem na implementação de medidas preventivas e de intervenções oportunas^(2)^.

Em vista disso, comunidades que vivem em áreas rurais, isoladas e/ou remotas, apresentam dificuldades no acesso aos serviços básicos, devido às suas particularidades biológicas, sociais, econômicas, intelectuais e políticas, como é o caso das populações ribeirinhas, que factualmente resistem e subsistem a muitas violações de direitos humanos^(3)^. Esse grupo é caracterizado pelo modo de vida específico, intrinsecamente associado à natureza e baseado na herança de saberes socioculturais que moldam o desenvolvimento das atividades cotidianas, as relações interpessoais e as práticas de cuidado em saúde^(4)^.

Na Amazônia brasileira, os ribeirinhos se originaram de processos históricos de miscigenação racial e interação entre povos distintos, que se tornaram vulneráveis diante de iniquidades sociais. Apesar dos direitos fundamentais de cidadania, estabelecidos na Constituição Federal de 1988^(5)^, e das ações de promoção à saúde apontadas na Política Nacional de Saúde Integral das Populações do Campo, da Floresta e das Águas (PNSIPCFA), publicada em 2011^(6)^, o cenário atual dos ribeirinhos é desafiador e principia o desamparo social. Essa realidade repercute nos conhecimentos e na habilidade de formar opiniões sobre temas variados, como intervenções em saúde adequadas, prevenção de doenças e promoção da saúde, envolvendo a competência para tomar decisões que visem manter ou melhorar a saúde e a qualidade de vida^(4,7)^. Assim, depreende-se o conceito de letramento em saúde (LS), que abrange a influência dos determinantes sociais e da aprendizagem prática^(7)^.

O LS promove a autonomia dos sujeitos para realizar escolhas informadas^(7)^. Segundo a Organização Mundial da Saúde (OMS), o LS abrange o conhecimento e as competências e habilidades individuais, de acordo com estruturas organizacionais e recursos disponíveis, os quais possibilitam que pessoas compreendam, avaliem e utilizem informações e serviços, e tenham acesso a eles, visando empreender ou manter condições de saúde com qualidade e bem-estar individual e coletivo^(8)^.

Nessa perspectiva, o letramento em saúde ambiental (LSA) consiste em uma das esferas do LS, que associa elementos e abordagens da saúde e das ciências ambientais e sociais, contribuindo para melhor entender questões relacionadas à exposição a riscos e à degradação ambiental, além das experiências do sujeito com o ambiente no qual reside. Também visa apreender a diversidade de competências e habilidades que as pessoas necessitam para buscar, compreender, avaliar e utilizar conhecimentos de saúde ambiental para tomar decisões conscientes, com a finalidade de proteger o meio ambiente e reduzir riscos que possam, em médio ou longo prazo, representar danos à saúde de indivíduos e grupos, configurando tema de estudo emergente^(9)^.

As disparidades no acesso à saúde, à educação e às tecnologias da informação e comunicação, entre ribeirinhos e a população em geral, repercutem no nível de LSA, uma vez que a disponibilidade, a propagação, o estímulo e a efetividade de práticas de ensino/educação e de saúde influenciam o modo como os indivíduos se comportam na relação com o meio ambiente^(4,10)^.

Considera-se que o LSA é, simultaneamente, um processo educativo e uma ferramenta de saúde pública, estando a saúde humana intimamente relacionada ao meio ambiente^(4,9)^. Nesse sentido, é preciso conhecer as competências e habilidades dos ribeirinhos acerca da relação entre saúde e ambiente no contexto locorregional, visando subsidiar intervenções de proteção socioambiental e valorizando as necessidades biopsicossociais^(9)^.

Diante da relevância do tema e da necessidade de compartilhá-lo, declarou-se o LSA de ribeirinhos da Amazônia paraense como objeto deste estudo. Para desenvolvê-lo, foram elaboradas as seguintes questões norteadoras: como se manifesta o LSA de ribeirinhos na Amazônia paraense? Quais fatores podem interferir nesse letramento? Como o LSA dos ribeirinhos pode repercutir em sua saúde? E como o LSA pode interferir no relacionamento dos ribeirinhos em torno do ambiente e das pessoas com as quais convivem? Assim, definiu-se como objetivo analisar o letramento em saúde ambiental e as suas repercussões no cotidiano de ribeirinhos da Amazônia paraense.

## MÉTODO

### Tipo de Estudo

Optou-se por estudo descritivo e qualitativo, tendo o LSA como base conceitual, no intuito de caracterizar, particularizar e compreender diversos elementos, tais como agentes sociais, comportamentos, crenças, decisões e opiniões que circulam nas relações humanas, como possibilita a abordagem qualitativa^(11)^. Sua redação foi guiada pelas orientações do *checklist COnsolidated criteria for REporting Qualitative research* (COREQ), valorizando três domínios, inerentes à equipe, ao desenho do estudo e ao conjunto de análises e resultados^(12)^.

É oportuno ressaltar que a abordagem quantitativa tem sido amplamente incorporada aos estudos de LS, com a utilização de instrumentos validados para mensurar níveis de compreensão e tomada de decisão. Entretanto, pelo objeto de estudo aqui declarado contemplar o LSA de uma população tradicional, optou-se pela abordagem qualitativa, tendo em vista que esse fenômeno exige uma compreensão ampliada do contexto, associada aos saberes tradicionais, às práticas cotidianas, às formas de comunicação, ao acesso à informação e às relações com o ambiente no qual os participantes se inserem. Tais aspectos ocorrem de forma complexa, particular e subjetiva, permeando-se por determinantes sociais da saúde e, portanto, não poderiam ser efetivamente acessados e apreendidos com escalas e medidas padronizadas.

Assim, entende-se que a abordagem qualitativa encontra respaldo teórico e metodológico, diante da necessidade de compreender os sentidos atribuídos à saúde ambiental e às suas potenciais repercussões no cotidiano dos sujeitos, gerando evidências que fortalecem os conhecimentos sobre a realidade investigada.

### Local

Ocorreu na Unidade Municipal de Saúde (UMS) da Ilha de Cotijuba, localizada no Distrito Administrativo de Outeiro (DAOUT), município de Belém, estado do Pará, Brasil. Gerida pela Secretaria Municipal de Saúde de Belém (SESMA), oferta serviços da Atenção Primária à Saúde (APS), associados à urgência e emergência, para atender às demandas da população.

Com cerca de 10.000 habitantes, a ilha tem passado por profundas transformações urbanas na última década, em virtude da proximidade com outros distritos de Belém, das atividades de turismo e da inserção de mais habitantes na dinâmica local, apesar das fragilidades na oferta de políticas públicas direcionadas a alguns setores, como saneamento básico, resíduos sólidos, vigilância sanitária e iluminação pública^(13,14)^, características que contribuíram para a escolha deste cenário.

Além disso, a UMS constitui o principal elo entre o sistema de saúde e a população local, favorecendo a consolidação de vínculos territoriais na comunidade. Trata-se de um cenário estratégico na APS, onde há maior proximidade com o cotidiano dos moradores e onde ocorrem ações para promover/recuperar a saúde e ações para prevenir doenças, favorecendo o contato com pessoas que buscam atendimento. Assim, possibilitou a escuta qualificada entre usuários e equipe de pesquisa, sendo elemento fundamental para investigar aspectos relacionados ao LSA.

### População e Critérios de Seleção

Participaram 29 ribeirinhos de ambos os sexos, com idade igual ou superior a 18 anos, residindo de forma permanente na ilha, independentemente de estarem matriculados na UMS. Excluiu-se apenas um ribeirinho, por apresentar hipossuficiência cognitiva, relatada por ele, a qual limitava sua capacidade comunicativa, não havendo recusas ou desistências. Assim, o termo “hipossuficiência cognitiva” foi utilizado para pré-estabelecer um critério de exclusão durante a elaboração do projeto, visando não comprometer o conteúdo das entrevistas.

Os participantes foram selecionados por conveniência, constituindo a amostra por saturação, identificada quando os dados obtidos se mostraram suficientes para apreender o objeto de estudo, não demandando novas entrevistas. Além disso, o quantitativo inserido atendeu à recomendação da literatura, que aponta de 20 a 30 participantes como intervalo satisfatório para alcançar objetivos em estudos qualitativos^(15)^.

Portanto, a saturação dos dados não foi considerada como elemento numérico, mas como processo analítico e reflexivo, que valoriza a análise do conteúdo simultânea à coleta dos dados, quando as entrevistas passam a apresentar recorrência de explicações e significados, sem acrescentar contribuições relevantes ao objeto de estudo, capazes de ampliar ou modificar a compreensão sobre o fenômeno investigado^(15)^.

### Coleta dos Dados

Para evitar possíveis vieses, os dados foram coletados por quatro pesquisadoras, devidamente orientadas e alinhadas para expressar postura similar durante as entrevistas. Visando assegurar o rigor na coleta, houve capacitação da equipe de pesquisa e supervisão do trabalho de campo por duas docentes. O alinhamento prévio das pesquisadoras teve o intuito de minimizar interferências que pudessem comprometer a qualidade e a autenticidade dos relatos, para não eliminar a dimensão subjetiva inerente ao processo de coleta, orientando-as a adotar linguagem acessível, clara e objetiva, para que os participantes compreendessem as perguntas a eles direcionadas. Esse alinhamento não buscou atribuir inflexibilidade técnica, mas assegurar o devido rigor metodológico, garantindo o respeito às narrativas e a exploração dos aspectos necessários para compreender suficientemente o objeto.

Aproximando-se do cenário, as pesquisadoras visitaram a UMS e se apresentaram aos profissionais que ali atuavam, dialogando sobre os objetivos e procedimentos da pesquisa. Conheceram a rotina dos serviços e agendaram, com o gestor local, dias e horários para realizar a coleta dos dados, que ocorreu em sala reservada nas dependências da unidade. Assim, os profissionais e os participantes não conheceram antecipadamente as pesquisadoras, e não estavam cientes das suas aspirações e trajetórias acadêmicas, nem do fato de que este estudo figurava como requisito para concluir as atividades do Programa Institucional de Bolsas de Iniciação Científica (PIBIC), razão pela qual as pesquisadoras dedicaram o tempo necessário para se aproximarem do cenário.

Os ribeirinhos foram abordados individualmente na sala de espera, antes ou depois dos atendimentos, sendo informados sobre o tema da pesquisa e convidados a participar. Visando garantir conforto e privacidade, os que aceitaram foram conduzidos à sala, na qual permaneceram somente o participante e uma pesquisadora. Os detalhes eram apresentados e as dúvidas esclarecidas com linguagem acessível, obtendo-se o aceite formal.

As entrevistas ocorreram no período de abril a julho de 2024, de forma individual, imediatamente após o aceite, sem demandar agendamento, e foram guiadas por um roteiro semiestruturado, elaborado pela equipe de pesquisa, contemplando dois eixos. Com nove perguntas objetivas, o primeiro eixo explorou o perfil sociodemográfico dos participantes, por meio das variáveis sexo, idade, raça/cor de pele, religião, nível de escolaridade, atividade profissional, renda familiar mensal, situação conjugal e pessoas com quem residiam. Com cinco perguntas subjetivas, desdobradas por meio do diálogo, o segundo eixo explorou conhecimentos acerca da saúde ambiental, onde e como os obtinham, se consideravam que o meio ambiente pode influenciar sua vida/saúde e de que maneira isso pode ocorrer, quais cuidados consideravam importantes para conservar o meio ambiente, se praticavam esses conhecimentos para prevenir doenças/agravos e como o faziam.

Considerando a robustez dos dados, não se identificou a necessidade de repetir entrevistas, assim como não foram utilizados diário de campo e técnicas de coleta adicionais. Embora não se tenha realizado teste piloto, destaca-se o cuidado da equipe de pesquisa em submeter o roteiro à avaliação prévia de três pesquisadores da área de enfermagem, com produção científica e qualificada em temas da saúde coletiva, os quais sugeriram alterações na primeira versão e referendaram a versão final.

Imprimiu-se uma via para cada participante. Assim, as respostas às perguntas do primeiro eixo foram registradas manualmente, enquanto as do segundo foram documentadas no formato MPEG-1/2 *audio layer* 3 (MP3), utilizando gravador digital (*Sony®* CD-PX240), com duração média de 12 minutos. É oportuno ressaltar que a duração média das respostas do segundo eixo foi suficiente para alcançar o objetivo e se aproxima de outras pesquisas que utilizaram técnicas de análise qualitativa com auxílio do mesmo *software* empregado neste estudo, as quais foram publicadas em periódicos científicos qualificados, informando duração média de oito, nove, dez e 18 minutos^(16–19)^.

Além disso, considerando o contexto sociocultural, a duração não foi utilizada como principal critério para avaliar a qualidade dos relatos, pois, em alguns casos, os participantes apresentavam acesso limitado à escolarização, o que pode ter influenciado a expressão verbal, mesmo com as investidas para extrair depoimentos com maior volume, por meio dos diálogos com as pesquisadoras. Em que pese a brevidade das respostas, houve clareza na expressão das subjetividades, contribuindo para a completude dos resultados, evidenciada na seção correspondente.

### Análise e Tratamento dos Dados

No *Microsoft Office Excel*®, versão 2024, os dados do perfil sociodemográfico foram organizados em planilha e analisados por estatística descritiva, gerando valores absolutos e relativos. As gravações foram transcritas na íntegra para constituir um *corpus* e submetê-lo à análise lexical no *software Interface de R pour les Analyses Multidimensionnelles de Textes et de Questionnaires* (IRaMuTeQ®), versão 0.7, *alpha* 2.

Criado por Pierre Ratinaud, o IRaMuTeQ® possibilita investigar criteriosamente dados textuais a partir da sua organização em arquivo único, sem formatação (arquivo .txt), e da separação em segmentos de texto (STs), ressaltando palavras frequentes e representativas, valores estatísticos, e a classificação gramatical das palavras. Inclui cinco modalidades analíticas: estatísticas textuais clássicas; especificidades e análise fatorial de correspondência; Classificação Hierárquica Descendente (CHD) ou método de Reinert, com aproveitamento satisfatório mínimo de 75%; análise de similitude; e nuvem de palavras^(20,21)^.

Entre as modalidades, utilizou-se a CHD, que pormenorizou o *corpus* em STs, originando classes lexicais, conforme a interdependência dos STs que as constituíram. Assim, as classes exprimiram significados particulares, apreendidos a partir da leitura dos STs, e foram ilustradas por um dendrograma, representação visual que evidencia o conjunto de palavras que as caracterizam semanticamente^(20,21)^.

O IRaMuTeQ® atribuiu valores estatísticos a essas palavras, entre os quais se destacam a frequência (F) de STs, que as apresentam nas classes, e o qui-quadrado (X^2^), que indica a força associativa das palavras, sendo representativas as que obtiveram p < 0,0001^(20,21)^. Optou-se por organizar as classes em eixos temáticos, que foram interpretados e discutidos à luz do LSA e da literatura científica pertinente e atualizada.

### Aspectos Éticos

Foram seguidos os preceitos éticos das Resoluções n° 466/2012 e nº 580/2018 do Conselho Nacional de Saúde e do Ministério da Saúde que, respectivamente, regulamentam pesquisas com seres humanos e suas especificidades éticas no Sistema Único de Saúde. Assim, o estudo foi autorizado pela SESMA e aprovado pelo Comitê de Ética em Pesquisa do curso de graduação em enfermagem da Universidade do Estado do Pará, sob Parecer nº 6.637.778, emitido em fevereiro de 2024. Os participantes leram e assinaram o Termo de Consentimento Livre e Esclarecido, sendo garantido sigilo das suas identidades com códigos alfanuméricos, compostos pela letra R, de “ribeirinho”, seguida por número arábico que indica a ordem das entrevistas.

## RESULTADOS

Entre os participantes, predominou o sexo feminino (n = 24; 82,76%), e a idade variou de 20 a 86 anos, destacando- se as faixas etárias de 20 a 24 (n = 5; 17,24%) e de 45 a 49 anos (n = 5; 17,24%), com média de 46,55. Quanto à raça/cor de pele, a maior proporção foi de pardos (n = 22; 75,86%), e no tocante à religião, houve predominância de evangélicos (n = 18; 62,07%). Os níveis de escolaridade mais expressivos foram ensino médio completo (n = 11; 37,93%) e ensino fundamental incompleto (n = 11; 37,93%). A maioria (n = 16; 55,17%) declarou exercer alguma atividade remunerada, ter renda familiar mensal de até um salário mínimo (n = 19; 65,52%), ser casada ou viver em união consensual (n = 17; 58,62%) e residir com uma ou mais pessoas (n = 27; 93,10%), sendo elas, em sua maioria, os filhos (n = 28; 96,55%).

No IRaMuTeQ®, o *corpus* se constitui por 29 textos, correspondentes ao total de entrevistas, gerando 552 STs, dos quais foram aproveitados 488 (88,41%). Identificaram-se 19.165 ocorrências (formas ou palavras), com 2.454 palavras distintas e 1.300 hápax, isto é, palavras com frequência um, representando 6,78% das ocorrências e 52,97% das palavras distintas. A média de palavras por ST foi, aproximadamente, 34,72. Foram estruturados dois *subcorpora* com sete classes lexicais, como ilustra o dendrograma da CHD (Figura 1). Seguindo a ordem de partição, a classe 1 compôs o primeiro *subcorpus*, e as classes 6, 7, 2, 4, 3 e 5 compuseram o segundo, de modo que as classes 6 e 7 foram geradas simultaneamente, assim como a dupla 2 e 4 e a dupla 3 e 5.

**Figura 1:** Dendrograma da Classificação Hierárquica Descendente. Belém, Pará, Brasil, 2024.

Essas classes foram organizadas em três eixos temáticos, sendo que o primeiro eixo se constituiu pelas classes 7, 6 e 5, denominadas “Significados atribuídos à saúde ambiental”, “Comportamentos diante do meio ambiente” e “Práticas para conservar o meio ambiente”. Compondo o segundo, as classes 3 e 2 foram intituladas “Influências do meio ambiente na saúde” e “Percepções sobre o contexto local”. Por sua vez, as classes 4 e 1 compuseram o terceiro, assim nomeadas: “Práticas para prevenir doenças e agravos” e “Desafios e modos de acessar informações sobre saúde e meio ambiente”. Tais eixos foram nomeados segundo o conteúdo das suas classes e são apresentados a seguir, com alguns excertos emblemáticos.

### Eixo Temático 1 – Saberes que Orientam os Modos de Lidar com o Meio Ambiente (Classes 7, 6 e 5)

Constituída por 78 STs (15,98% do *corpus*) e 20 palavras representativas (p < 0,0001), a classe 7 abrange os modos de compreender a saúde ambiental a partir dos saberes sobre ela, dos processos experienciados e da realidade em que viviam os participantes. Nesse sentido, palavras como “limpo” (X^2^ = 110,55), “manter” (X^2^ = 68,98) e “bem” (X^2^ = 54,40) evidenciam relações entre a noção de limpeza e a projeção imagética de um ambiente saudável e propício ao bem-estar dos indivíduos que nele interagem:

*Saúde ambiental, para mim, é o meio ambiente onde eu vivo. É necessário cuidar da coleta de lixo e um pouco de tudo, porque eu acho que o ambiente tem que estar limpo e organizado.* (R9)

*O meio ambiente pode influenciar a minha saúde e o bem-estar da minha vida. Por isso, eu penso que devemos manter a limpeza para ter um ambiente limpo.* (R25)

Observou-se a interligação entre saúde ambiental, qualidade de vida e felicidade dos participantes, atrelando o bem-estar físico e mental à moradia em ambientes sem poluição e com a natureza preservada:

*Saúde ambiental é viver bem em um lugar de paz e sem poluição.* (R21)

*Se você não respirar um ar puro, você não estará bem, por isso a natureza é um ambiente que precisa estar sempre puro, limpo e saudável, para você se sentir feliz.* (R24)

Percebeu-se que o conhecimento dos participantes acerca do conceito de saúde ambiental era incipiente, visto que o relacionaram aos aspectos visíveis do ambiente, fazendo com que não explicitassem grande parte dos elementos que o constituem, como a interação ecológica entre seres vivos, as condições aérea e climática da natureza e as relações sociais construídas nesse contexto:

*A* [falta de] *saúde ambiental, no meu ponto de vista, é ter muito lixo na água e no solo.* (R4)

*Saúde ambiental é o meio ambiente sem lixo na rua, preservar a natureza e os rios.* (R15)

Formada por 61 STs (12,50% do *corpus*) e 20 palavras representativas (p < 0,0001), a classe 6 elucida os comportamentos dos participantes diante do meio ambiente, construindo a ideia de que agiam conforme as possibilidades da realidade que vivenciavam. A palavra “lixo” (X^2^ = 49,74), que apresentou o segundo maior qui-quadrado da classe, associada ao verbo “descartar” (X^2^ = 50,13) e à palavra “jogo” (X^2^ = 48,92), anuncia práticas inadequadas de separação, armazenamento e descarte de resíduos sólidos:

*Quando se trata de lixo,* [em casa] *não há separação, e todo tipo de lixo é jogado na rua.* (R12)

*Descarto, na rua, o lixo que não posso queimar, para não acumular e nem jogar no quintal.* (R15)

A ausência de um sistema adequado para coleta de lixo favorecia a adoção de alternativas para solucionar o acúmulo de resíduos domésticos, podendo gerar repercussões negativas à saúde e ao meio ambiente:

*Queimamos o lixo para não amontoar e não trazer insetos.* (R10)

*Queimo o lixo porque, às vezes, o carro de coleta passa e não recolhe todo o lixo da rua. Para evitar colocar na frente de casa e atrair mosquitos, eu prefiro queimar.* (R24)

Elaborada com 76 STs (15,57% do *corpus*) e nove palavras representativas (p < 0,0001), a classe 5 expressa práticas cotidianas para conservar o ambiente. Nesse sentido, as palavras “desmatar” (X^2^ = 35,58), “queimado” (X^2^ = 32,34) e “cuidar” (X^2^ = 30,91) remetem, ao mesmo tempo, à preocupação e à necessidade de preservar o meio ambiente, incluindo a proteção de florestas e rios. Além disso, remetem à noção de que não aderir às práticas de degradação ambiental ajuda a melhorar a natureza:

*É importante não desmatar o meio ambiente. Quando falo em meio ambiente, é a floresta, incluindo os peixes e tudo sobre os rios. É importante não despejar lixo e sacos plásticos.* (R1)

*Eu acho que todos devem pensar em cuidar do meio ambiente e, por isso, as pessoas não deveriam fazer queimadas, para ajudar a diminuir a poluição do ar, para o meio ambiente ficar limpo.* (R19)

A repetição do verbo “depender” (X^2^ = 15,95), em STs dessa classe, atribui sentido à importância da preservação ambiental, correlacionada com o sustento e a saúde de populações ribeirinhas no que tange às fontes de alimentação e renda, configuradas, especialmente, pela agricultura e pela pesca:

*Se o ser humano não tiver cuidado com o meio ambiente, pode causar doenças. Por isso, temos que cuidar dos rios para não morrerem, porque dependemos disso para pescar o camarão e o peixe.* (R1)

*Onde eu morava, fui acostumada a cuidar bastante das árvores e não desmatar, porque muitas frutas vêm das árvores, principalmente da mangueira e do açaizeiro, até porque dependemos economicamente disso hoje em dia.* (R3)

### Eixo Temático 2 – Perspectivas sobre a Relação entre Meio Ambiente e Saúde (Classes 3 e 2)

Em sua formação, a classe 3 apresentou 79 STs (16,19% do *corpus*), sendo a maior classe, e 20 palavras representativas (p < 0,0001), entre as quais destacam-se “influenciar” (X^2^ = 66,47), “saúde” (X^2^ = 59,41), “afetar” (X^2^ = 54,28) e “fumaça” (X^2^ = 51,61). Tais palavras expressam o reconhecimento da relação entre meio ambiente e condições de saúde, demonstrando a vulnerabilidade da saúde humana diante da degradação ambiental e a poluição do ambiente como aspecto que facilita a transmissão de doenças:

*Eu faço queimadas e acho que isso deixa a minha saúde mais vulnerável no meio ambiente, porque as queimadas prejudicam a saúde por causa da fumaça.* (R12)

*Considero que a poluição e o desmatamento causam muitos impactos na vida e na saúde. O ar que eu respiro é afetado pela fumaça das queimadas e por combustíveis e fábricas que poluem muito.* (R18)

Foi possível apreender relatos que elucidam a compreensão dos participantes sobre os diversos fatores contidos no ambiente que os predispõem ao adoecimento, com a possibilidade de agravar esse cenário, mediante a disseminação de doenças na comunidade:

*O ambiente ruim prejudica a nossa saúde e pode causar várias doenças, como a doença de rato, que é a leptospirose, e muitas outras.* (R3)

*Existem coisas que podem afetar a nossa saúde, como a água parada, a dengue, o lixo e as queimadas, já que a fumaça faz mal pra mim, porque tenho bronquite crônica, que adquiri devido a isso* [referindo-se às queimadas]*.* (R5)

Composta por 77 STs (15,78% do *corpus*) e 13 palavras representativas (p < 0,0001), a classe 2 evidenciou o senso de pertencimento dos ribeirinhos ao contexto local, por meio de elementos associados à residência própria e às atividades ocupacionais, mas também à poluição ambiental resultante das ações dos moradores, acrescidas do descarte de resíduos, inadequadamente praticado por visitantes, retratando os impactos do turismo no meio ambiente. Palavras como “ilha” (X^2^ = 50,58), “praia” (X^2^ = 49,72) e “água” (X^2^ = 21,09) denotam essa perspectiva:

*Com a chegada das férias, as coisas pioram muito, porque as pessoas chegam na ilha, bagunçam tudo e vão embora, deixando a rua horrível. Mas as pessoas que vivem aqui não conseguem sair, porque a casa delas está aqui.* (R5)

*Nós trabalhamos na praia e moramos em frente a ela, então estamos o tempo todo coletando lixo, porque as pessoas que vêm de fora, e até mesmo as pessoas daqui, usam o local e deixam lixo.* (R12)

Nesse contexto, alguns depoimentos revelaram sensação de abandono por parte da administração pública e falta de saneamento básico na ilha, o que poderia comprometer a saúde e a qualidade de vida dos moradores. Essa situação era agravada por problemas com a coleta de lixo, que resultavam na contaminação ambiental e na degradação de recursos naturais, corroborando a necessidade de políticas públicas efetivas para suprir as demandas da população e preservar o ambiente:

*Aqui, na ilha, não temos tratamento de esgoto e nem tratamento de água. A comunidade está tendo problemas na coleta de lixo e, por isso, o ambiente está contaminado.* (R12)

*Nós somos muito abandonados pelo poder público, pelos governos e por todos que administram nossa ilha. Embora tenha muito turismo, a ilha é abandonada pelo poder público.* (R27)

### Eixo Temático 3 – Protagonismo dos Ribeirinhos e Responsabilidade Socioambiental (Classes 4 e 1)

Elaborada com 44 STs (9,02% do *corpus*) e 20 palavras representativas (p < 0,0001), a classe 4 é a menor. Retrata os saberes e aprendizados adquiridos entre os ribeirinhos para manter a saúde e aplicar medidas que os protegessem de possíveis doenças e agravos, como evidenciado pelas palavras “cuidado” (X^2^ = 44,09), “prevenir” (X^2^ = 42,70), “contaminado” (X^2^ = 40,90), “contrair” (X^2^ = 31,03) e “doença” (X^2^ = 22,02). Tais medidas configuravam práticas que emergiam de informações prévias, muitas vezes incipientes e que eram obtidas e compartilhadas a partir das experiências e dos saberes tradicionais:

*Em casa, tenho cuidado com o lixo, enquanto outras pessoas não têm e deixam no meio da rua. Então, o chorume vai para o lençol freático e usamos a água contaminada do poço. Por isso, a minha preocupação é adquirirmos doenças que venham dessa água.* (R10)

*Para me prevenir de doenças, me afasto de pessoas que estão possivelmente contaminadas.* (R16)

As palavras “lavar” (X^2^ = 62,71) e “água empoçada” (X^2^ = 61,30) apresentam os dois maiores qui-quadrado da classe, refletindo a preocupação dos participantes com a higiene ambiental e o controle de vetores para que microrganismos e insetos não se reproduzissem em locais quentes e úmidos, propiciados pelas características do clima local. Nessa perspectiva, também se mostraram preocupados em evitar água parada para controlar o adoecimento por dengue:

*Tiro as folhas de açaí que caem para não deixar água empoçada, viro as vasilhas para não deixar água acumular, tenho cuidado com a água que fica armazenada e tampo tudo. Acho que isso é bom, porque vai prevenir a dengue e outras doenças.* (R15)

*O açaí é a prova que você pode contrair doenças. Por exemplo, se o barbeiro* [inseto] *pousar no açaí e você não lavar ou não tomar cuidado, você pega a doença. Meu sogro pegou, porque ele não se cuidava.* (R22)

Composta por 73 STs (14,96% do *corpus*) e 27 palavras representativas (p < 0,0001), a classe 1 aborda alguns desafios e os meios pelos quais os ribeirinhos acessavam informações relativas à saúde e ao meio ambiente. Palavras como “informação” (X^2^ = 330,05), “acesso” (X^2^ = 180,64) e “dificuldade” (X^2^ = 116,90) retratam importantes lacunas, vivenciadas por alguns participantes (n = 13; 44,83%), no acesso a informações sobre o tema ao longo da vida, a partir da falta de recursos e infraestrutura e do déficit na qualidade do conteúdo disponibilizado a essa população, distanciando-a em relação às fontes confiáveis:

*Quem não tem celular, televisão e internet, não consegue ter acesso a informações, e há pessoas em Cotijuba que não têm.* (R5)

*É muito difícil ter acesso a informações sobre saúde por meios de comunicação e, quando é possível, a informação é superficial.* (R12)

Entre os meios de acesso disponíveis à outra parcela dos participantes (n = 22; 75,86%), as palavras “internet” (X^2^ = 137,22), “televisão” (X^2^ = 106,25), “jornal” (X^2^ = 52,13) e “celular” (X^2^ = 40,37) apontam as fontes digitais empregadas no cotidiano para obter informações. Assim, identificou-se o quanto elas ajudavam a disseminar saberes e práticas, podendo influenciar a tomada de decisão e promover a autonomia na busca por novos conhecimentos. Percebeu-se, também, a importância atribuída aos profissionais de saúde que, durante visitas domiciliares, contribuíam com explicações sobre o tema:

*Pesquiso também na internet e no YouTube*®*. Hoje em dia, está muito avançado. Há muitos meios* [de comunicação] *e muitas informações boas sobre o meio ambiente.* (R9)

*Vejo muitas informações sobre isso pela internet, pelo celular e pelos jornais que passam na televisão, além de ter informações* [fornecidas] *pelos médicos e agentes comunitários de saúde, que sempre estão ajudando e explicando.* (R18)

Evidenciou-se o termo “palestra” (X^2^ = 28,72), demonstrando o anseio por mais informações, com orientações adequadas, que possam ser incorporadas à realidade local. Isso apontou a necessidade de iniciativas que eduquem e sensibilizem os moradores:

*Com certeza, encontro várias dificuldades para ter acesso à informação. Seria bom se tivéssemos uma forma de ter acesso na ilha, como uma palestra, mas não temos.* (R13)

[Os profissionais de saúde] *poderiam entregar um folheto marcando um horário e local para explicarem a nós, porque não sabemos sobre essas informações.* (R25)

## DISCUSSÃO

Os resultados evidenciam que as percepções dos ribeirinhos sobre saúde ambiental se relacionaram aos elementos do seu cotidiano e foram influenciadas pelos determinantes sociais da saúde, que delineiam seus modos de vida e a condição ambiental vivenciada^(22)^.

As limitações de escolaridade dos ribeirinhos podem influenciar o desenvolvimento econômico e repercutir no acesso a bens de consumo, como recursos para mobilidade até os serviços de saúde geograficamente distantes, configurando barreiras para promover tanto o acesso às informações sobre saúde e meio ambiente quanto a compreensão adequada acerca delas^(23)^. Isso interfere nos modos pelos quais essa população gerencia suas condições de vida e saúde em um ambiente marcado por especificidades territoriais, conjuntura que envolve o LSA^(10,24)^.

No contexto dessas reflexões, organizou-se a discussão de acordo com a lógica dos resultados, estruturando-a por meio das seções apresentadas abaixo.

### Eixo Temático 1 – Saberes que Orientam os Modos de Lidar com o Meio Ambiente (Classes 7, 6 e 5)

Sabe-se que o contexto cultural dos ribeirinhos molda concepções e comportamentos diante das relações interpessoais e do ambiente em que vivem, influenciando a convivência direta com a natureza, permeada por elementos biopsicossociais que caracterizam esse grupo^(25)^. Assim, suas interpretações revelaram uma compreensão prática sobre saúde ambiental, associando- a aos esforços cotidianos pontuais para manter o ambiente sem descarte inadequado de resíduos sólidos, fundamentando a construção de uma consciência ambiental resultante de experiências prévias.

Compreensões como essa transcendem a ideia de sujeira e poluição, ao atribuírem sensação de bem-estar aos que transitam e interatuam em um espaço bem tratado, o que expressa uma forma de convivência harmoniosa com o meio ambiente, na medida em que os elementos naturais são concebidos como componentes da identidade cultural e dos modos de vida. Nessa perspectiva, afetos podem ser atribuídos ao ambiente e associados à valorização de práticas e saberes locais^(25)^.

Isso demonstra que um ambiente limpo proporciona condições favoráveis à saúde, destacando vínculos entre práticas ambientais e prevenção de riscos e agravos, o que favorece a promoção de um ambiente saudável, alinhado à qualidade de vida. Todavia, o limitado conhecimento que os participantes expressaram ao conceituar saúde ambiental demonstrou uma visão reducionista, com ênfase nos resíduos sólidos e nos corpos hídricos, pois a realidade que enfrentavam assinalava condições sanitárias precárias, confrontada com o fato de que dependiam dos recursos naturais.

Essa limitação pode ser entendida ao considerar que o LSA se associa a diversos fatores, incluindo características sociodemográficas, fontes de informação e práticas cotidianas^(10)^, contexto em que os níveis insatisfatórios de educação ofertada às populações em áreas rurais culminam por estabelecer desvantagens para que obtenham informações confiáveis sobre aspectos relacionados à saúde. Tais desvantagens dificultam a boa gestão de cuidados, a prevenção de agravos e a tomada de decisão informada^(3)^.

Além disso, a infraestrutura deficiente dos serviços de saneamento básico colaborava para os ribeirinhos adotarem medidas alternativas, no intuito de evitar o acúmulo de resíduos em locais de convívio social, que poderiam abrigar roedores e servir de criadouros para mosquitos, sobretudo em recipientes que acumulavam água, aumentando os riscos para contrair dengue e outras patologias relacionadas à depredação ambiental. Para garantir saúde e segurança no domicílio, relataram-se iniciativas assentadas na queima de resíduos a céu aberto. No entanto, é oportuno destacar que a emissão de poluentes na atmosfera pode reduzir a imunidade e aumentar a ocorrência de alergias, infecções e processos inflamatórios em diferentes estruturas anatomofisiológicas, como nariz e garganta^(26)^.

### Eixo Temático 2 – Perspectivas sobre a Relação entre Meio Ambiente e Saúde (Classes 3 e 2)

Os participantes reconheceram os impactos da degradação ambiental na saúde humana, ao associarem o desflorestamento, a emissão de gases poluentes e a urbanização ao desenvolvimento de patologias respiratórias vivenciadas por eles^(27)^. Essa realidade desvela condições ambientais desfavoráveis como fatores que determinam a saúde das populações, especialmente de comunidades vulneráveis e que carecem de saneamento básico, incluindo desde a falta de estrutura para esgotamento sanitário e distribuição de água potável até a destinação inadequada de resíduos^(28)^.

Na tentativa de esclarecer formas de adoecimento resultantes da poluição ambiental, os participantes rememoraram condições pregressas para apontar fatores que os tornavam predispostos a adoecer, caracterizando o ambiente mal cuidado como facilitador da ocorrência e disseminação de doenças infecciosas^(23)^. Nesse processo, os sujeitos recorrem às suas experiências para explicar fatos, esforçando-se para encontrar respostas à fonte dos problemas, ancorando-se, principalmente, no senso comum^(29)^.

As percepções sobre o contexto local evidenciaram a representação do meio ambiente como espaço de sustento e pertencimento, no qual as ações irresponsáveis de moradores e visitantes foram atreladas à poluição da água, do solo e do ar, gerando desequilíbrios ambientais. Em cenários como esse, o turista é percebido como novo elemento, que deixa marcas nos espaços já ocupados pelos atores locais^(30)^.

Ações externas e caracterizadas pela degradação ambiental tendem a repercutir na forma como os moradores atribuem sentido e agem diante do ambiente e das pessoas do seu convívio. Reconhece-se que o envolvimento de populações tradicionais com o turismo configura um fenômeno em crescimento, mas é necessário priorizar o cenário, a identidade cultural e a manutenção dos recursos naturais, valorizando os saberes e as cosmologias particulares de cada grupo^(30)^.

Também se identificou o sentimento de abandono por parte das autoridades públicas, que não atendiam de maneira eficaz às necessidades relativas ao saneamento básico, incluindo a coleta de resíduos. Historicamente, comunidades em zonas rurais são destituídas de recursos que estimulem a melhoria da qualidade de vida, englobando o acesso à educação, à saúde e ao saneamento^(23)^. O Relatório de Metas de Desenvolvimento do Milênio, publicado em 2015, expôs os contrastes das condições de vida entre a zona urbana e a zona rural, apontando que o acesso à água potável e ao saneamento foi, respectivamente, quatro e três vezes menor em zonas rurais quando comparadas com zonas urbanas. Além disso, a chance de crianças não frequentarem escolas foi quatro vezes maior em zonas rurais quando comparadas com zonas urbanas, repercutindo nos indicadores de morbidade e mortalidade^(31)^.

Nesse contexto, os Objetivos de Desenvolvimento Sustentável expressam a qualidade de vida como direito universal, devendo ser garantido a todos, e, para isso, cada pessoa necessita de oportunidades de acesso ao conhecimento em saúde que seja compatível com seu nível de entendimento, tornando-a capaz de tomar decisões adequadas no cotidiano^(22)^.

### Eixo Temático 3 – Protagonismo dos Ribeirinhos e Responsabilidade Socioambiental (Classes 4 e 1)

Os ribeirinhos convivem com um histórico de mudanças climáticas no ambiente em que habitam, sobretudo com a enchente e vazante dos rios. Essa realidade culmina em saberes populares que norteiam práticas para lidar com iniquidades características da região^(32)^.

Comportamentos declarados por essas pessoas evidenciam o reconhecimento de ações básicas de higiene e preservação da natureza, com a finalidade de mitigar riscos para o adoecimento, enfatizando o que é descrito no LSA acerca das competências e habilidades para compreender, avaliar e utilizar informações sobre saúde ambiental^(10,26)^. A adesão de medidas sanitárias foi justificada a partir da necessidade de conservar o meio ambiente, incluindo fauna, flora, águas superficiais e subterrâneas, solo e ar, concepção que pode resultar em melhores indicadores de saúde, devido à maior proteção contra doenças e agravos relacionados à degradação, como reiterado na literatura^(26)^.

Desigualdades no acesso a informações confiáveis fragilizam o processo de aprendizagem dos ribeirinhos sobre o tema, limitando sua habilidade para compreender e aplicar intervenções que visem manter a saúde e conservar o ambiente. Disparidades como essas são geralmente influenciadas por carência de recursos financeiros e tecnológicos, infraestrutura precária, escassez de ações educativas, e reduzida acessibilidade a serviços básicos, como serviços de saúde e educação^(33)^.

Apesar dos desafios que enfrentavam para obter informações sobre saúde e meio ambiente por iniciativas próprias, evidenciou-se o crescente uso da internet como recurso que permite acessar conteúdos diversos e que pode alcançar grande parcela da população, como apontado na literatura^(7)^. Assim, meios de comunicação digital podem aproximar grupos geograficamente isolados às informações sobre esse e outros temas, auxiliando- os a buscar soluções práticas e fortalecer a autonomia para tomar decisões^(32)^.

Outro aspecto importante é a visita domiciliar realizada por profissionais de saúde, que constitui valiosa oportunidade de interação entre a equipe e a comunidade, favorecendo o compartilhamento de informações, o esclarecimento de dúvidas e a promoção de práticas preventivas, de acordo com as necessidades biopsicossociais de cada indivíduo. Nas visitas, além das condições de vida e saúde, é possível avaliar detalhadamente as condições ambientais, possibilitando identificar riscos potenciais e fornecer orientações contextualizadas, especialmente para aqueles que não possuem meios de acesso facilitado à informação ou apresentam dificuldades para compreender^(23)^.

Expressou-se o anseio por mais informações, indicando que os participantes reconheciam a necessidade de melhor entender como suas ações afetavam não apenas as suas condições de vida e saúde, mas também a saúde coletiva e a qualidade dos recursos disponíveis no ambiente. Assim, é fundamental promover iniciativas educacionais dialógicas e emancipadoras para capacitar a população e facilitar seu engajamento, sensibilizando-a para criar redes colaborativas com o objetivo de fortalecer a saúde e a qualidade de vida, em consonância com a preservação do meio ambiente^(33,34)^.

As limitações deste estudo se referem ao fato de ter sido desenvolvido em cenário específico, com características econômicas, políticas, socioculturais e territoriais particulares, restringindo a amplitude geográfica dos dados. Todavia, o conjunto dos resultados e as reflexões que dele emanaram podem contribuir significativamente com as áreas de enfermagem e saúde coletiva, ao fornecer subsídios para comparar e interpretar resultados de outras pesquisas em contextos similares, aprimorar ações de cuidado no contexto ribeirinho em relação à saúde ambiental, e desenvolver políticas públicas, programas educacionais e outras iniciativas direcionadas à promoção da saúde e à proteção ambiental.

## CONCLUSÃO

Demonstrou-se que o LSA de ribeirinhos da Amazônia paraense envolve a atribuição de significados ao cenário que os cerca, a adoção de práticas para cuidar do meio ambiente e da saúde, e o acesso a informações sobre o tema. Portanto, é fundamental para que os ribeirinhos compreendam como suas ações repercutem na saúde individual e coletiva, envolvendo a inter-relação entre práticas de higiene, saneamento básico e proteção ambiental. No entanto, fatores como acesso limitado à informação, condições socioeconômicas desfavoráveis e carência de iniciativas educacionais qualificadas podem dificultar o LSA, interferindo na habilidade de tomar decisões diante das condições do ambiente.

As repercussões do LSA na saúde dos ribeirinhos podem ser significativas, contribuindo para prevenir o adoecimento e promover um estilo de vida saudável. Nessa perspectiva, sensibilizar quanto à necessidade de preservar o meio ambiente pode fortalecer nos ribeirinhos o relacionamento com seu entorno, fomentando o senso de comunidade para estabelecer corresponsabilidades. Portanto, investir na educação e na qualificação das ações executadas é essencial para empoderar essas pessoas e, assim, torná-las agentes ativos na preservação do ambiente e na melhoria das suas condições de vida e saúde.

Com base nos dados, entende-se que será possível gerar outros conhecimentos científicos, obtidos com robustez, por meio das atividades de pesquisa na enfermagem e em áreas correlatas, para embasar ou fomentar a criação, implementação e efetividade de políticas públicas direcionadas às necessidades dessas populações. Ao serem cultural e socialmente adaptadas, essas políticas podem contribuir para a equidade no acesso à saúde e para a preservação ambiental em territórios historicamente vulnerabilizados.

Nesse contexto, os profissionais de enfermagem, sobretudo os enfermeiros, poderão agir de modo a promover saúde, a fim de que pessoas e grupos acessem, compreendam, avaliem e utilizem conhecimentos sobre saúde e meio ambiente para tomar decisões com consciência crítica, empoderando-se nos cuidados consigo e com o meio que os cerca, tornando-se agentes de mudança em suas comunidades. Isso evidencia o papel do enfermeiro como educador, devido ao contexto social das populações ribeirinhas, marcado por baixa escolaridade e difícil acesso à informação.

## Data Availability

Os dados que dão suporte a este estudo podem ser disponibilizados mediante solicitação ao autor correspondente.

# Translation: Alfabetización en salud ambiental entre residentes ribereños de la región amazónica de Pará

**DOI:** 10.1590/1980-220X-REEUSP-2025-0015es

**Authors:** Élida Fernanda Rêgo de Andrade, Sandy Isabelly Osório de Sousa, Sarah Souza de Carvalho, Débora de Cássia Quaresma Silva, Erlon Gabriel Rego de Andrade, Ana Kedma Correa Pinheiro, Ivaneide Leal Ataíde Rodrigues, Paula Gisely Costa Silva, Laura Maria Vidal Nogueira

**Affiliations:** 1 Universidade do Estado do Pará, Centro de Ciências Biológicas e da Saúde, Escola de Enfermagem Magalhães Barata, Belém, PA, Brasil.; 2 Universidade do Estado do Pará, Escola de Enfermagem Magalhães Barata, Programa de Pós-Graduação em Enfermagem, Belém, PA, Brasil.; 3 Universidade Federal do Rio de Janeiro, Escola de Enfermagem Anna Nery, Programa de Pós-Graduação em Enfermagem, Rio de Janeiro, RJ, Brasil.

**Keywords:** Alfabetización en Salud, Salud Ambiental, Población Rural, Salud Rural, Salud Pública

## Abstract

**Objetivo:**

Analizar la alfabetización en salud ambiental y su impacto en la vida cotidiana de una comunidad ribereña de la región amazónica de Pará.

**Método:**

Estudio cualitativo descriptivo realizado con 29 residentes ribereños en la Unidad Municipal de Salud de la Isla de Cotijuba en Belém, Pará, Brasil. Los datos se recopilaron entre abril y julio de 2024 mediante entrevistas individuales semiestructuradas. El *corpus* se sometió a análisis léxico mediante el *software Interface de R pour les Analyses Multidimensionnelles de Textes et de Questionnaires* 0.7/*alpha* 2, utilizando la Clasificación Jerárquica Descendente.

**Resultados:**

Se identificaron 552 segmentos textuales, de los cuales 488 (88,41%) fueron utilizados, generándose siete clases léxicas, organizadas en tres ejes temáticos, que apuntaron conocimientos para el trato con el medio ambiente, la relación entre medio ambiente y salud, y el protagonismo y responsabilidad socioambiental de los residentes ribereños.

**Conclusión:**

La alfabetización en salud ambiental en residentes ribereños implica los significados que atribuyen al entorno que las rodea, la adopción de prácticas de cuidado y el acceso a información sobre el tema. Esto es esencial para que comprendan cómo sus acciones impactan la salud individual y colectiva.

## INTRODUCCIÓN

La salud ambiental valora la correlación entre los factores del medio natural y la salud humana, condicionada a las modificaciones antrópicas en el medio ambiente, ya que éstas influyen y determinan la calidad de vida del ser humano^(1)^. En este sentido, las condiciones ambientales son determinantes fundamentales de la salud, mientras que las desigualdades en el acceso a la información y a los recursos interfieren en la implementación de medidas preventivas e intervenciones oportunas^(2)^.

En vista de ello, las comunidades que viven en zonas rurales, aisladas y/o remotas tienen dificultades para acceder a los servicios básicos, debido a sus particularidades biológicas, sociales, económicas, intelectuales y políticas, como es el caso de las poblaciones ribereñas, que de hecho resisten y subsisten a muchas violaciones de los derechos humanos^(3)^. Este grupo se caracteriza por un modo de vida específico, intrínsecamente asociado a la naturaleza y basado en la herencia de conocimientos socioculturales que moldean el desarrollo de las actividades cotidianas, las relaciones interpersonales y las prácticas de cuidado en salud^(4)^.

En la Amazonia brasileña, los ribereños tienen su origen en procesos históricos de mestizaje racial e interacción entre pueblos distintos, que se han vuelto vulnerables ante las desigualdades sociales. A pesar de los derechos fundamentales de ciudadanía establecidos en la Constitución Federal de 1988^(5)^ y de las acciones de promoción de la salud señaladas en la Política Nacional de Salud Integral de las Poblaciones del Campo, de la Selva y de las Aguas (PNSIPCFA en la sigle en portugués), publicada en 2011^(6)^, el panorama actual de los ribereños es desafiante y da lugar al desamparo social. Esta realidad repercute en los conocimientos y en la capacidad de formar opiniones sobre temas variados, como las intervenciones en salud adecuadas, la prevención de enfermedades y la promoción de la salud, lo que implica la competencia para tomar decisiones que visen mantener o mejorar la salud y la calidad de vida^(4,7)^. De ahí se deriva el concepto de alfabetización en salud (AS), que abarca la influencia de los determinantes sociales y el aprendizaje práctico^(7)^.

La AS promueve la autonomía de los sujetos para tomar decisiones informadas^(7)^. Según la Organización Mundial de la Salud (OMS), la AS abarca los conocimientos, las competencias y las habilidades individuales, de acuerdo con las estructuras organizativas y los recursos disponibles, que permiten a las personas comprender, evaluar y utilizar la información y los servicios, y tener acceso a ellos, con el fin de alcanzar o mantener condiciones de salud con calidad y bienestar individual y colectivo^(8)^.

En esta perspectiva, la alfabetización en salud ambiental (ASA) consiste en una de las esferas de la AS, que asocia elementos y enfoques de la salud y las ciencias ambientales y sociales, contribuyendo a comprender mejor las cuestiones relacionadas con la exposición a los riesgos y la degradación ambiental, además de las experiencias del sujeto con el medio ambiente en el que reside. También busca captar la diversidad de competencias y habilidades que las personas necesitan para buscar, comprender, evaluar y utilizar los conocimientos de salud ambiental para tomar decisiones conscientes, con el fin de proteger el medio ambiente y reducir los riesgos que, a medio o largo plazo, puedan representar daños para la salud de individuos y grupos, lo que constituye un tema de estudio emergente^(9)^.

Las disparidades en el acceso a la salud, la educación y las tecnologías de la información y la comunicación entre los ribereños y la población en general repercuten en el nivel de ASA, ya que la disponibilidad, la difusión, el estímulo y la eficacia de las prácticas de enseñanza/educación y salud influyen en la forma en que los individuos se comportan en relación con el medio ambiente^(4,10)^.

Se considera que ASA es, al mismo tiempo, un proceso educativo y una herramienta de salud pública, ya que la salud humana está íntimamente relacionada con el medio ambiente^(4,9)^. En este sentido, es necesario conocer las competencias y habilidades de los ribereños sobre la relación entre salud y medio ambiente en el contexto local y regional, con el fin de subsidiar intervenciones de protección socioambiental y valorizar las necesidades biopsicosociales^(9)^.

Dada la relevancia del tema y la necesidad de compartirlo, se declaró ASA de los ribereños de la Amazonia paraense como objeto de este estudio. Para desarrollarlo, se elaboraron las siguientes preguntas orientadoras: ¿cómo se manifiesta la ASA de los ribereños en la Amazonia paraense? ¿Qué factores pueden interferir en esta alfabetización? ¿Cómo puede repercutir la ASA de los ribereños en su salud? ¿Y cómo puede interferir la ASA en la relación de los ribereños con el medio ambiente y con las personas con las que conviven? Así, se definió como objetivo analizar la alfabetización en salud ambiental y sus repercusiones en la vida cotidiana de los ribereños de la Amazonia paraense.

## MÉTODO

### Tipo de Estudio

Se optó por un estudio descriptivo y cualitativo, teniendo como base conceptual la ASA, con el fin de caracterizar, particularizar y comprender diversos elementos, tales como agentes sociales, comportamientos, creencias, decisiones y opiniones que circulan en las relaciones humanas, tal y como lo permite el enfoque cualitativo^(11)^. Su redacción se ha guiado por las orientaciones de la lista de verificación *COnsolidated criteria for REporting Qualitative research* (COREQ), valorando tres ámbitos inherentes al equipo, al diseño del estudio y al conjunto de análisis y resultados^(12)^.

Cabe destacar que el enfoque cuantitativo se ha incorporado ampliamente a los estudios de AS, con el uso de instrumentos validados para medir los niveles de comprensión y toma de decisiones. Sin embargo, dado que el objeto de estudio aquí declarado contempla la ASA de una población tradicional, se optó por el enfoque cualitativo, teniendo en cuenta que este fenómeno exige una comprensión ampliada del contexto, asociada a los conocimientos tradicionales, las prácticas cotidianas, las formas de comunicación, el acceso a la información y las relaciones con el entorno en el que se insertan los participantes. Estos aspectos se presentan de forma compleja, particular y subjetiva, impregnados de determinantes sociales de la salud y, por lo tanto, no podrían ser efectivamente accedidos y comprendidos con escalas y medidas estandarizadas.

Así, se entiende que el enfoque cualitativo encuentra respaldo teórico y metodológico, ante la necesidad de comprender los significados atribuidos a la salud ambiental y sus posibles repercusiones en la vida cotidiana de los sujetos, generando evidencias que fortalecen el conocimiento sobre la realidad investigada.

### Lugar

Se llevó a cabo en la Unidad Municipal de Salud (UMS) de la isla de Cotijuba, situada en el Distrito Administrativo de Outeiro (DAOUT), municipio de Belém, estado de Pará, Brasil. Gestionada por la Secretaría Municipal de Salud de Belém (SESMA), ofrece servicios de Atención Primaria de Salud (APS), asociados a la urgencia y la emergencia, para atender las demandas de la población.

Con unos 10 000 habitantes, la isla ha experimentado profundas transformaciones urbanas en la última década, debido a su proximidad a otros distritos de Belém, a las actividades turísticas y a la incorporación de más habitantes a la dinámica local, a pesar de las deficiencias en la oferta de políticas públicas dirigidas a algunos sectores, como el saneamiento básico, los residuos sólidos, la vigilancia sanitaria y el alumbrado público^(13,14)^, características que contribuyeron a la elección de este escenario.

Además, la UMS constituye el principal vínculo entre el sistema de salud y la población local, lo que favorece la consolidación de los lazos territoriales en la comunidad. Se trata de un escenario estratégico en la APS, donde hay una mayor proximidad con la vida cotidiana de los residentes y donde se llevan a cabo acciones para promover/recuperar la salud y acciones para prevenir enfermedades, favoreciendo el contacto con las personas que buscan atención. De este modo, se ha facilitado la escucha cualificada entre los usuarios y el equipo de investigación, lo que ha sido un elemento fundamental para investigar aspectos relacionados con la ASA.

### Población y Criterios de Selección

Participaron 29 ribereños de ambos sexos, con edad igual o superior a 18 años, residentes de forma permanente en la isla, independientemente de estar inscritos en la UMS.

Solo se excluyó a un ribereño, por presentar insuficiencia cognitiva, según él mismo informó, lo que limitaba su capacidad comunicativa, sin que hubiera rechazos ni renuncias. Así, el término «insuficiencia cognitiva» se utilizó para preestablecer un criterio de exclusión durante la elaboración del proyecto, con el fin de no comprometer el contenido de las entrevistas.

Los participantes fueron seleccionados por conveniencia, constituyendo la muestra por saturación, identificada cuando los datos obtenidos resultaron suficientes para comprender el objeto de estudio, sin necesidad de nuevas entrevistas. Además, el número de participantes se ajustó a la recomendación de la literatura, que señala entre 20 y 30 participantes como intervalo satisfactorio para alcanzar los objetivos en estudios cualitativos^(15)^.

Por lo tanto, la saturación de los datos no se consideró como un elemento numérico, sino como un proceso analítico y reflexivo, que valora el análisis del contenido simultáneo a la recolección de datos, cuando las entrevistas comienzan a presentar explicaciones y significados recurrentes, sin añadir contribuciones relevantes al objeto de estudio, capaces de ampliar o modificar la comprensión sobre el fenómeno investigado^(15)^.

### Recolección de Datos

Para evitar posibles sesgos, los datos fueron recolectados por cuatro investigadoras, debidamente orientadas y alineadas para expresar una postura similar durante las entrevistas. Con el fin de garantizar el rigor en la recolección, se capacitó al equipo de investigación y se supervisó el trabajo de campo por parte de dos docentes. La alineación previa de las investigadoras tuvo como objetivo minimizar las interferencias que pudieran comprometer la calidad y la autenticidad de los relatos, sin eliminar la dimensión subjetiva inherente al proceso de recolección, orientándolas a adoptar un lenguaje accesible, claro y objetivo, para que los participantes comprendieran las preguntas que se les formulaban. Esta coordinación no buscaba imponer una rigidez técnica, sino garantizar el rigor metodológico y el respeto por las narrativas, así como explorar los aspectos necesarios para comprender suficientemente el objeto de estudio.

Para acercarse al escenario, las investigadoras visitaron la UMS y se presentaron a los profesionales que trabajaban allí, dialogando sobre los objetivos y procedimientos de la investigación. Conocieron la rutina de los servicios y acordaron con el administrador local los días y horarios para realizar la recolección de datos, que se llevó a cabo en una sala reservada en las instalaciones de la unidad. De este modo, los profesionales y los participantes no conocían de antemano a las investigadoras, ni eran conscientes de sus aspiraciones y trayectorias académicas, ni del hecho de que este estudio era un requisito para concluir las actividades del Programa Institucional de Becas de Iniciación Científica (PIBIC), razón por la cual las investigadoras dedicaron el tiempo necesario para acercarse al escenario.

Se abordó a los ribereños de forma individual en la sala de espera, antes o después de las consultas, se les informó sobre el tema de la investigación y se les invitó a participar. Con el fin de garantizar la comodidad y la privacidad, los que aceptaron fueron conducidos a la sala, en la que solo permanecieron el participante y una investigadora. Se presentaron los detalles y se aclararon las dudas con un lenguaje accesible, obteniéndose la aceptación formal.

Las entrevistas se realizaron entre abril y julio de 2024, de forma individual, inmediatamente después de la aceptación, sin necesidad de cita previa, y se guiaron por un guion semiestructurado, elaborado por el equipo de investigación, que contemplaba dos ejes. Con nueve preguntas objetivas, el primer eje exploró el perfil sociodemográfico de los participantes, a través de las variables sexo, edad, raza/color de piel, religión, nivel de escolaridad, actividad profesional, ingreso familiar mensual, situación conyugal y personas con quienes residían. Con cinco preguntas subjetivas, desarrolladas a través del diálogo, el segundo eje exploró los conocimientos sobre salud ambiental, dónde y cómo los obtenían, si consideraban que el medio ambiente podía influir en su vida/salud y de qué manera, qué cuidados consideraban importantes para conservar el medio ambiente, si ponían en práctica esos conocimientos para prevenir enfermedades/afecciones y cómo lo hacían.

Teniendo en cuenta la solidez de los datos, no se identificó la necesidad de repetir las entrevistas, ni se utilizaron diarios de campo ni técnicas de recolección adicionales. Aunque no se realizó una prueba piloto, cabe destacar el cuidado del equipo de investigación al someter el guion a la evaluación previa de tres investigadores del área de enfermería, con producción científica y cualificados en temas de salud colectiva, quienes sugirieron cambios en la primera versión y refrendaron la versión final.

Se imprimió una copia para cada participante. Así, las respuestas a las preguntas del primer eje se registraron manualmente, mientras que las del segundo se documentaron en formato MPEG-1/2 *audio layer* 3 (MP3), utilizando una grabadora digital (*Sony*® CD-PX240), con una duración media de 12 minutos. Cabe destacar que la duración media de las respuestas del segundo eje fue suficiente para alcanzar el objetivo y se aproxima a otras investigaciones que utilizaron técnicas de análisis cualitativo con la ayuda del mismo *software* empleado en este estudio, las cuales fueron publicadas en revistas científicas calificadas, informando una duración media de ocho, nueve, diez y 18 minutos^(16-19)^.

Además, considerando el contexto sociocultural, la duración no se utilizó como criterio principal para evaluar la calidad de los relatos, ya que, en algunos casos, los participantes tenían acceso limitado a la escolarización, lo que puede haber influido en la expresión verbal, incluso con los esfuerzos por extraer testimonios más extensos, a través de diálogos con las investigadoras. A pesar de la brevedad de las respuestas, hubo claridad en la expresión de las subjetividades, lo que contribuyó a la exhaustividad de los resultados, como se evidencia en la sección correspondiente.

### Análisis y Tratamiento de los Datos

En *Microsoft Office Excel*®, versión 2024, los datos del perfil sociodemográfico se organizaron en una hoja de cálculo y se analizaron mediante estadística descriptiva, generando valores absolutos y relativos. Las grabaciones se transcribieron íntegramente para constituir un *corpus* y someterlo a análisis léxico en el *software Interface de R pour les Analyses Multidimensionnelles de Textes et de Questionnaires* (IRaMuTeQ®), versión 0.7, *alpha* 2.

Creado por Pierre Ratinaud, el IRaMuTeQ® permite investigar minuciosamente datos textuales a partir de su organización en un único archivo, sin formato (archivo .txt), y de su separación en segmentos de texto (STs), resaltando palabras frecuentes y representativas, valores estadísticos y la clasificación gramatical de las palabras. Incluye cinco modalidades analíticas: estadísticas textuales clásicas; especificidades y análisis factorial de correspondencia; Clasificación Jerárquica Descendente (CJD) o método de Reinert, con un aprovechamiento mínimo satisfactorio del 75%; análisis de similitud; y nube de palabras^(20,21)^.

Entre las modalidades, se utilizó la CJD, que detalló el *corpus* en STs, dando lugar a clases léxicas, según la interdependencia de los STs que las constituían. Así, las clases expresaron significados particulares, extraídos de la lectura de los STs, y se ilustraron mediante un dendrograma, representación visual que muestra el conjunto de palabras que las caracterizan semánticamente^(20,21)^.

El IRaMuTeQ® asignó valores estadísticos a estas palabras, entre los que destacan la frecuencia (F) de los STs que las presentan en las clases y el chi-cuadrado (X^2^), que indica la fuerza asociativa de las palabras, siendo representativas aquellas que obtuvieron p < 0,0001^(20,21)^. Se optó por organizar las clases en ejes temáticos, que se interpretaron y discutieron a la luz dla ASA y de la literatura científica pertinente y actualizada.

### Aspectos Éticos

Se siguieron los preceptos éticos de las Resoluciones n.º 466/2012 y n.° 580/2018 del Consejo Nacional de Salud y del Ministerio de Salud que, respectivamente, regulan la investigación con seres humanos y sus especificidades éticas en el Sistema Único de Salud.

Así, el estudio fue autorizado por la SESMA y aprobado por el Comité de Ética en Investigación del curso de licenciatura en enfermería de la *Universidade do Estado do Pará*, mediante el Dictamen n.º 6.637.778, emitido en febrero de 2024. Los participantes leyeron y firmaron el Término de Consentimiento Libre y Esclarecido, garantizándose el secreto de sus identidades con códigos alfanuméricos, compuestos por la letra R, de «ribereño», seguida de un número arábigo que indica el orden de las entrevistas.

## RESULTADOS

Entre los participantes predominó el sexo femenino (n = 24; 82,76%), y la edad varió entre 20 y 86 años, destacando los grupos de edad de 20 a 24 (n = 5; 17,24%) y de 45 a 49 años (n = 5; 17,24%), con una media de 46,55. En cuanto a la raza/color de piel, la mayor proporción fue de mestizos (n = 22; 75,86%), y en cuanto a la religión, predominaron los evangélicos (n = 18; 62,07%). Los niveles de escolaridad más significativos fueron secundaria completa (n = 11; 37,93%) y primaria incompleta (n = 11; 37,93%). La mayoría (n = 16; 55,17%) declaró ejercer alguna actividad remunerada, tener ingresos familiares mensuales de hasta un salario mínimo (n = 19; 65,52%), estar casada o vivir en unión consensual (n = 17; 58,62%) y residir con una o más personas (n = 27; 93,10%), siendo en su mayoría hijos (n = 28; 96,55%).

En el IRaMuTeQ®, el *corpus* está constituido por 29 textos, correspondientes al total de entrevistas, generando 552 STs, de los cuales se aprovecharon 488 (88,41%). Se identificaron 19.165 ocurrencias (formas o palabras), con 2.454 palabras distintas y 1.300 hapax, es decir, palabras con frecuencia uno, lo que representa el 6,78% de las ocurrencias y el 52,97% de las palabras distintas. El promedio de palabras por ST fue de aproximadamente 34,72. Se estructuraron dos *subcorpus* con siete clases léxicas, como ilustra el dendrograma de la CJD (Figura 1). Siguiendo el orden de partición, la clase 1 compuso el primer *subcorpus*, y las clases 6, 7, 2, 4, 3 y 5 compusieron el segundo, de modo que las clases 6 y 7 se generaron simultáneamente, al igual que el dúo 2 y 4 y el dúo 3 y 5.

**Figura 1:** Dendrograma de la Clasificación Jerárquica Descendente. Belém, Pará, Brasil, 2024.

Estas clases se organizaron en tres ejes temáticos, siendo el primero el constituido por las clases 7, 6 y 5, denominadas «Significados atribuidos a la salud ambiental», «Comportamientos frente al medio ambiente» y «Prácticas para conservar el medio ambiente». El segundo eje está compuesto por las clases 3 y 2, denominadas «Influencias del medio ambiente en la salud» y «Percepciones sobre el contexto local». Por su parte, las clases 4 y 1 componen el tercer eje, denominadas «Prácticas para prevenir enfermedades y afecciones» y «Desafíos y formas de acceder a la información sobre salud y medio ambiente». Estos ejes se nombraron según el contenido de sus clases y se presentan a continuación, con algunos extractos emblemáticos.

### Eje Temático 1 – Conocimientos que Orientan las Formas de Relacionarse con el Medio Ambiente (Clases 7, 6 y 5)

Constituida por 78 STs (15,98% del *corpus*) y 20 palabras representativas (p < 0,0001), la clase 7 abarca las formas de entender la salud ambiental a partir de los conocimientos sobre ella, los procesos vividos y la realidad en la que vivían los participantes. En este sentido, palabras como «limpio» (X^2^ = 110,55), «mantener» (X^2^ = 68,98) y «bien» (X^2^ = 54,40) evidencian relaciones entre la noción de limpieza y la proyección imaginaria de un entorno saludable y propicio para el bienestar de las personas que interactúan en él:

*Para mí, la salud ambiental es el medio ambiente en el que vivo. Es necesario cuidar la recolección de basura y un poco de todo, porque creo que el medio ambiente tiene que estar limpio y organizado.* (R9)

*El medio ambiente puede influir en mi salud y en mi bienestar. Por eso, creo que debemos mantener la limpieza para tener un medio ambiente limpio.* (R25)

Se observó la interconexión entre la salud ambiental, la calidad de vida y la felicidad de los participantes, vinculando el bienestar físico y mental con la vivienda en entornos sin contaminación y con la naturaleza preservada:

*La salud ambiental es vivir bien en un lugar tranquilo y sin contaminación.* (R21)

*Si no respiras aire puro, no estarás bien, por eso la naturaleza es un entorno que debe estar siempre puro, limpio y saludable, para que te sientas feliz.* (R24)

Fue constatado que el conocimiento de los participantes sobre el concepto de salud ambiental era incipiente, ya que lo relacionaban con aspectos visibles del medio ambiente, lo que les impedía explicitar gran parte de los elementos que lo constituyen, como la interacción ecológica entre los seres vivos, las condiciones atmosféricas y climáticas de la naturaleza y las relaciones sociales construidas en ese contexto:

*La* [falta de] *salud ambiental, en mi punto de vista, es tener mucha basura en el agua y en el suelo.* (R4)

*La salud ambiental es el medio ambiente sin basura en la calle, preservar la naturaleza y los ríos.* (R15)

Formada por 61 STs (12,50% del *corpus*) y 20 palabras representativas (p < 0,0001), la clase 6 aclara los comportamientos de los participantes frente al medio ambiente, construyendo la idea de que actuaban según las posibilidades de la realidad que vivían. La palabra «basura» (X^2^ = 49,74), que presentó el segundo mayor chi-cuadrado de la clase, asociada al verbo «descartar» (X^2^ = 50,13) y a la palabra «juego» (X^2^ = 48,92), anuncia prácticas inadecuadas de separación, almacenamiento y eliminación de residuos sólidos:

*Cuando se trata de basura,* [en casa] *no se separa y todo tipo de basura se tira a la calle.* (R12)

*Tiro a la calle la basura que no puedo quemar, para que no se acumule ni se tire al patio.* (R15)

La ausencia de un sistema adecuado de recolección de basura favorecía la adopción de alternativas para solucionar la acumulación de residuos domésticos, lo que podía generar repercusiones negativas para la salud y el medio ambiente:

*Quemamos la basura para que no se amontone y no atraiga insectos.* (R10)

*Quemo la basura porque, a veces, el camión de recolección pasa y no recoge toda la basura de la calle. Para evitar ponerla frente a la casa y atraer mosquitos, prefiero quemarla.* (R24)

Elaborada con 76 STs (15,57% del *corpus*) y nueve palabras representativas (p < 0,0001), la clase 5 expresa prácticas cotidianas para conservar el medio ambiente. En este sentido, las palabras «deforestar» (X^2^ = 35,58), «quemado» (X^2^ = 32,34) y «cuidar» (X^2^ = 30,91) remiten, al mismo tiempo, a la preocupación y a la necesidad de preservar el medio ambiente, incluida la protección de los bosques y los ríos. Además, remiten a la noción de que no adherirse a las prácticas de degradación ambiental ayuda a mejorar la naturaleza:

*Es importante no deforestar el medio ambiente. Cuando hablo de medio ambiente, me refiero al bosque, incluyendo los peces y todo lo que hay en los ríos. Es importante no tirar basura ni bolsas de plástico.* (R1)

*Creo que todos debemos pensar en cuidar el medio ambiente y, por eso, la gente no debería quemar, para ayudar a reducir la contaminación del aire, para que el medio ambiente esté limpio.* (R19)

La repetición del verbo «depender» (X^2^ = 15,95) en las ST de esta clase atribuye sentido a la importancia de la preservación ambiental, correlacionada con el sustento y la salud de las poblaciones ribereñas en lo que respecta a las fuentes de alimentación e ingresos, configuradas, especialmente, por la agricultura y la pesca:

*Si el ser humano no cuida el medio ambiente, puede causar enfermedades. Por eso, tenemos que cuidar los ríos para que no mueran, porque dependemos de ellos para pescar camarones y pescado.* (R1)

*Donde yo vivía, estaba acostumbrada a cuidar mucho los árboles y a no talarlos, porque muchos frutos provienen de los árboles, principalmente del mango y del palma de açai, ya que hoy en día dependemos económicamente de ellos.* (R3)

### Eje Temático 2 – Perspectivas sobre la Relación entre Medio Ambiente y Salud (Clases 3 y 2)

En su formación, la clase 3 presentó 79 STs (16,19% del *corpus*), siendo la clase más numerosa, y 20 palabras representativas (p < 0,0001), entre las que destacan «influir» (X^2^ = 66,47), «salud» (X^2^ = 59,41), «afectar» (X^2^ = 54,28) y «humo» (X^2^ = 51,61). Estas palabras expresan el reconocimiento de la relación entre el medio ambiente y las condiciones de salud, lo que demuestra la vulnerabilidad de la salud humana ante la degradación ambiental y la contaminación del medio ambiente como un aspecto que facilita la transmisión de enfermedades:

*Hago quemadas y creo que eso hace que mi salud sea más vulnerable en el medio ambiente, porque los quemados perjudican la salud por el humo.* (R12)

*Considero que la contaminación y la deforestación causan muchos impactos en la vida y en la salud. El aire que respiro está afectado por el humo de los quemados y por los combustibles y las fábricas que contaminan mucho.* (R18)

Se pudieron recabar relatos que aclaran la comprensión de los participantes sobre los diversos factores presentes en el medio ambiente que los predisponen a enfermarse, con la posibilidad de agravar este escenario mediante la propagación de enfermedades en la comunidad:

*El mal ambiente perjudica nuestra salud y puede causar varias enfermedades, como la enfermedad del ratón, que es la leptospirosis, y muchas otras.* (R3)

*Hay cosas que pueden afectar nuestra salud, como el agua estancada, el dengue, la basura y las quemaduras, ya que el humo me hace daño porque tengo bronquitis crónica, que contraje debido a eso* [refiriéndose a las quemaduras]*.* (R5)

Compuesta por 77 STs (15,78% del *corpus*) y 13 palabras representativas (p < 0,0001), la clase 2 evidenció el sentido de pertenencia de los ribereños al contexto local, a través de elementos asociados a la residencia propia y a las actividades ocupacionales, pero también a la contaminación ambiental resultante de las acciones de los residentes, a lo que se suma el descarte inadecuado de residuos por parte de los visitantes, retratando los impactos del turismo en el medio ambiente. Palabras como «isla» (X^2^ = 50,58), «playa» (X^2^ = 49,72) y «agua» (X^2^ = 21,09) denotan esta perspectiva:

*Con la llegada de las vacaciones, las cosas empeoran mucho, porque la gente llega a la isla, lo desordenan todo y se van, dejando la calle horrible. Pero la gente que vive aquí no puede irse, porque su casa está aquí.* (R5)

*Trabajamos en la playa y vivimos frente a ella, así que estamos todo el tiempo recogiendo basura, porque la gente que viene de fuera, e incluso la gente de aquí, usa el lugar y deja basura.* (R12)

En este contexto, algunos testimonios revelaron una sensación de abandono por parte de la administración pública y la falta de saneamiento básico en la isla, lo que podría comprometer la salud y la calidad de vida de los residentes. Esta situación se veía agravada por problemas con la recolección de basura, que provocaban la contaminación ambiental y la degradación de los recursos naturales, lo que corroboraba la necesidad de políticas públicas eficaces para satisfacer las demandas de la población y preservar el medio ambiente:

*Aquí, en la isla, no tenemos tratamiento de aguas residuales ni tratamiento de agua. La comunidad tiene problemas con la recolección de basura y, por eso, el medio ambiente está contaminado.* (R12)

*Estamos muy abandonados por el gobierno, por las autoridades y por todos los que administran nuestra isla. Aunque hay mucho turismo, la isla está abandonada por el gobierno.* (R27)

### Eje Temático 3: Protagonismo de los Ribereños y Responsabilidad Socioambiental (Clases 4 y 1)

Elaborada con 44 STs (9,02% del *corpus*) y 20 palabras representativas (p < 0,0001), la clase 4 es la más pequeña. Refleja los conocimientos y aprendizajes adquiridos entre los ribereños para mantener la salud y aplicar medidas que los protejan de posibles enfermedades y afecciones, como lo evidencian las palabras «cuidado» (X^2^ = 44,09), «prevenir» (X^2^ = 42,70), «contaminado» (X^2^ = 40,90), «contraer» (X^2^ = 31,03) y «enfermedad» (X^2^ = 22,02). Dichas medidas configuraban prácticas que surgían de información previa, a menudo incipiente, y que se obtenía y compartía a partir de las experiencias y los conocimientos tradicionales:

*En casa, tengo cuidado con la basura, mientras que otras personas no lo tienen y la dejan en medio de la calle. Entonces, el lodo llega al agua subterránea y utilizamos el agua contaminada del pozo. Por eso, mi preocupación es contraer enfermedades que provengan de esa agua.* (R10)

*Para prevenirme de enfermedades, me alejo de personas que puedan estar contaminadas.* (R16)

Las palabras «lavar» (X^2^ = 62,71) y «agua empozada» (X^2^ = 61,30) presentan los dos chi-cuadrado más altos de la clase, lo que refleja la preocupación de los participantes por la higiene ambiental y el control de vectores para que los microorganismos y los insectos no se reproduzcan en lugares cálidos y húmedos, propicios por las características del clima local. En esta perspectiva, también se mostraron preocupados por evitar el agua estancada para controlar la enfermedad del dengue:

*Quito las hojas de açaí que caen para que no se encharque el agua, doy la vuelta a los recipientes para que no se acumule el agua, tengo cuidado con el agua que se almacena y lo tapo todo. Creo que esto es bueno, porque previene el dengue y otras enfermedades.* (R15)

*El açaí es la prueba de que se pueden contraer enfermedades. Por ejemplo, si la vinchuca* [insecto] *se posa en el açaí y no te lavas o no tienes cuidado, contraes la enfermedad. Mi suegro la contrajo porque no se cuidaba.* (R22)

Compuesta por 73 STs (14,96% del *corpus*) y 27 palabras representativas (p < 0,0001), la clase 1 aborda algunos retos y los medios por los que los ribereños accedían a la información relativa a la salud y al medio ambiente. Palabras como «información» (X^2^ = 330,05), «acceso» (X^2^ = 180,64) y «dificultad» (X^2^ = 116,90) reflejan importantes carencias experimentadas por algunos participantes (n = 13; 44,83%) en el acceso a información sobre el tema a lo largo de la vida, debido a la falta de recursos e infraestructura y al déficit en la calidad del contenido disponible para esta población, lo que la aleja de fuentes confiables:

*Quien no tiene celular, televisión ni internet, no puede acceder a la información, y hay personas en Cotijuba que no tienen.* (R5)

*Es muy difícil acceder a información sobre salud a través de los medios de comunicación y, cuando es posible, la información es superficial.* (R12)

Entre los medios de acceso disponibles para otra parte de los participantes (n = 22; 75,86%), las palabras «internet» (X^2^ = 137,22), «televisión» (X^2^ = 106,25), «periódico» (X^2^ = 52,13) y «celular» (X^2^ = 40,37) señalan las fuentes digitales utilizadas en la vida cotidiana para obtener información. Así, se identificó en qué medida ayudaban a difundir conocimientos y prácticas, pudiendo influir en la toma de decisiones y promover la autonomía en la búsqueda de nuevos conocimientos. También se percibió la importancia atribuida a los profesionales de la salud que, durante las visitas domiciliarias, contribuían con explicaciones sobre el tema:

*También busco en Internet y en YouTube*®*. Hoy en día está muy avanzado. Hay muchos medios* [de comunicación] *y mucha información buena sobre el medio ambiente.* (R9)

*Veo mucha información sobre esto en Internet, en el celular y en los periódicos que salen en la televisión, además de tener información* [proporcionada] *por los médicos y los agentes comunitarios de salud, que siempre están ayudando y explicando.* (R18)

Se evidenció el término «conferencia» (X^2^ = 28,72), lo que demuestra el deseo de obtener más información, con orientaciones adecuadas, que puedan incorporarse a la realidad local. Esto apuntó a la necesidad de iniciativas que eduquen y sensibilicen a los residentes:

*Sin duda, encuentro varias dificultades para acceder a la información. Sería bueno que tuviéramos alguna forma de acceder a ella en la isla, como una conferencia, pero no la tenemos.* (R13)

[Los profesionales de la salud] *podrían entregar un folleto con una fecha y un lugar para explicárnoslo, porque no sabemos nada sobre estos temas.* (R25)

## DISCUSIÓN

Los resultados evidencian que las percepciones de los ribereños sobre la salud ambiental se relacionan con elementos de su vida cotidiana y están influenciadas por los determinantes sociales de la salud, que configuran sus modos de vida y las condiciones ambientales que experimentan^(22)^.

Las limitaciones educativas de los ribereños pueden influir en el desarrollo económico y repercutir en el acceso a bienes de consumo, como los recursos para desplazarse a los servicios de salud geográficamente distantes, lo que configura barreras para promover tanto el acceso a la información sobre salud y medio ambiente como la comprensión adecuada de la misma^(23)^. Esto interfiere en las formas en que esta población gestiona sus condiciones de vida y salud en un entorno marcado por especificidades territoriales, una coyuntura que involucra a la ASA^(10,24)^.

En el contexto de estas reflexiones, se organizó la discusión de acuerdo con la lógica de los resultados, estructurándola a través de las secciones que se presentan a continuación.

### Eje Temático 1 – Conocimientos que Orientan las Formas de Relacionarse con el Medio Ambiente (Clases 7, 6 y 5)

Se sabe que el contexto cultural de los ribereños moldea las concepciones y los comportamientos frente a las relaciones interpersonales y al entorno en el que viven, influyendo en la convivencia directa con la naturaleza, impregnada de elementos biopsicosociales que caracterizan a este grupo^(25)^. Así, sus interpretaciones revelaron una comprensión práctica de la salud ambiental, asociándola a los esfuerzos cotidianos puntuales para mantener el medio ambiente sin vertidos inadecuados de residuos sólidos, lo que fundamenta la construcción de una conciencia ambiental resultante de experiencias previas.

Comprensiones como esta trascienden la idea de suciedad y contaminación, al atribuir una sensación de bienestar a quienes transitan e interactúan en un espacio bien tratado, lo que expresa una forma de convivencia armoniosa con el medio ambiente, en la medida en que los elementos naturales se conciben como componentes de la identidad cultural y los modos de vida. Desde esta perspectiva, se pueden atribuir afectos al medio ambiente y asociarlos a la valoración de las prácticas y los conocimientos locales^(25)^.

Esto demuestra que un medio ambiente limpio proporciona condiciones favorables para la salud, destacando los vínculos entre las prácticas ambientales y la prevención de riesgos y daños, lo que favorece la promoción de un medio ambiente saludable, en consonancia con la calidad de vida. Sin embargo, el conocimiento limitado que los participantes expresaron al conceptualizar la salud ambiental demostró una visión reduccionista, con énfasis en los residuos sólidos y los cuerpos de agua, ya que la realidad que enfrentaban señalaba condiciones sanitarias precarias, confrontadas con el hecho de que dependían de los recursos naturales.

Esta limitación puede entenderse si se considera que la ASA se asocia a diversos factores, entre ellos las características sociodemográficas, las fuentes de información y las prácticas cotidianas^(10)^, contexto en el que los niveles insatisfactorios de educación ofrecidos a las poblaciones de las zonas rurales terminan por establecer desventajas para que obtengan información fiable sobre aspectos relacionados con la salud. Estas desventajas dificultan la buena gestión de la atención, la prevención de enfermedades y la toma de decisiones informadas^(3)^.

Además, la deficiente infraestructura de los servicios de saneamiento básico contribuía a que los ribereños adoptaran medidas alternativas para evitar la acumulación de residuos en lugares de convivencia social, que podían albergar roedores y servir de criaderos de mosquitos, sobre todo en recipientes que acumulaban agua, lo que aumentaba el riesgo de contraer dengue y otras enfermedades relacionadas con la depredación ambiental. Para garantizar la salud y la seguridad en los hogares, se informaron iniciativas basadas en la quema de residuos al aire libre. Sin embargo, cabe destacar que la emisión de contaminantes a la atmósfera puede reducir la inmunidad y aumentar la aparición de alergias, infecciones y procesos inflamatorios en diferentes estructuras anatomo-fisiológicas, como la nariz y la garganta^(26)^.

### Eje Temático 2 – Perspectivas sobre la Relación entre Medio Ambiente y Salud (Clases 3 y 2)

Los participantes reconocieron los impactos de la degradación ambiental en la salud humana, al asociar la deforestación, la emisión de gases contaminantes y la urbanización con el desarrollo de patologías respiratorias que ellos mismos padecen^(27)^. Esta realidad revela que las condiciones ambientales desfavorables son factores que determinan la salud de las poblaciones, especialmente de las comunidades vulnerables y que carecen de saneamiento básico, desde la falta de infraestructura para el alcantarillado y la distribución de agua potable hasta la eliminación inadecuada de residuos^(28)^.

En un intento por aclarar las formas de enfermedad resultantes de la contaminación ambiental, los participantes recordaron condiciones pasadas para señalar los factores que los predisponían a enfermarse, caracterizando el entorno mal cuidado como facilitador de la aparición y propagación de enfermedades infecciosas^(23)^. En este proceso, los sujetos recurren a sus experiencias para explicar los hechos, esforzándose por encontrar respuestas al origen de los problemas, basándose principalmente en el sentido común^(29)^.

Las percepciones sobre el contexto local evidenciaron la representación del medio ambiente como un espacio de sustento y pertenencia, en el que las acciones irresponsables de los residentes y visitantes se relacionaron con la contaminación del agua, el suelo y el aire, generando desequilibrios ambientales. En escenarios como este, el turista es percibido como un elemento nuevo, que deja huellas en los espacios ya ocupados por los actores locales.

Las acciones externas caracterizadas por la degradación ambiental tienden a repercutir en la forma en que los residentes atribuyen sentido y actúan ante el medio ambiente y las personas con las que conviven. Se reconoce que la implicación de las poblaciones tradicionales en el turismo constituye un fenómeno en crecimiento, pero es necesario dar prioridad al entorno, la identidad cultural y la conservación de los recursos naturales, valorando los conocimientos y las cosmologías particulares de cada grupo^(30)^.

También se identificó el sentimiento de abandono por parte de las autoridades públicas, que no atendían de manera eficaz las necesidades relacionadas con el saneamiento básico, incluida la recolección de residuos. Históricamente, las comunidades de las zonas rurales carecen de recursos que estimulen la mejora de la calidad de vida, incluyendo el acceso a la educación, la salud y el saneamiento^(23)^. El Informe sobre los Objetivos de Desarrollo del Milenio, publicado en 2015, expuso los contrastes entre las condiciones de vida en las zonas urbanas y rurales, señalando que el acceso al agua potable y al saneamiento era, respectivamente, cuatro y tres veces menor en las zonas rurales que en las urbanas. Además, la probabilidad de que los niños no asistieran a la escuela era cuatro veces mayor en las zonas rurales que en las urbanas, lo que se reflejaba en los indicadores de morbilidad y mortalidad^(31)^.

En este contexto, los Objetivos de Desarrollo Sostenible expresan la calidad de vida como un derecho universal que debe garantizarse a todos y, para ello, cada persona necesita oportunidades de acceso a conocimientos en salud que sean compatibles con su nivel de comprensión, lo que le permite tomar decisiones adecuadas en la vida cotidiana^(22)^.

### Eje Temático 3: Protagonismo de los Ribereños y Responsabilidad Socioambiental (Clases 4 y 1)

Los ribereños conviven con un historial de cambios climáticos en el entorno en el que viven, sobre todo con las crecidas y bajadas de los ríos. Esta realidad culmina en conocimientos populares que orientan las prácticas para hacer frente a las desigualdades características de la región^(32)^.

Los comportamientos declarados por estas personas evidencian el reconocimiento de acciones básicas de higiene y preservación de la naturaleza, con el fin de mitigar los riesgos de enfermedad, enfatizando lo descrito en la ASA sobre las competencias y habilidades para comprender, evaluar y utilizar la información sobre salud ambiental^(10,26)^. La adhesión a las medidas sanitarias se justificó por la necesidad de conservar el medio ambiente, incluyendo la fauna, la flora, las aguas superficiales y subterráneas, el suelo y el aire, concepción que puede dar lugar a mejores indicadores de salud, debido a una mayor protección contra las enfermedades y los agravios relacionados con la degradación, como se reitera en la literatura^(26)^.

Las desigualdades en el acceso a información confiable debilitan el proceso de aprendizaje de los ribereños sobre el tema, limitando su capacidad para comprender y aplicar intervenciones destinadas a mantener la salud y conservar el medio ambiente. Disparidades como estas suelen estar influenciadas por la falta de recursos financieros y tecnológicos, la precaria infraestructura, la escasez de acciones educativas y la reducida accesibilidad a servicios básicos, como los servicios de salud y educación^(33)^.

A pesar de los retos a los que se enfrentaban para obtener información sobre salud y medio ambiente por iniciativa propia, se evidenció el creciente uso de Internet como recurso que permite acceder a contenidos diversos y que puede llegar a una gran parte de la población, como se señala en la literatura^(7)^. Así, los medios de comunicación digitales pueden acercar a grupos geográficamente aislados a la información sobre este y otros temas, ayudándoles a buscar soluciones prácticas y a fortalecer su autonomía para tomar decisiones^(32)^.

Otro aspecto importante son las visitas domiciliarias realizadas por profesionales de la salud, que constituyen una valiosa oportunidad de interacción entre el equipo y la comunidad, favoreciendo el intercambio de información, la aclaración de dudas y la promoción de prácticas preventivas, de acuerdo con las necesidades biopsicosociales de cada individuo. En las visitas, además de las condiciones de vida y salud, es posible evaluar en detalle las condiciones ambientales, lo que permite identificar riesgos potenciales y proporcionar orientación contextualizada, especialmente para aquellos que no tienen medios de acceso fácil a la información o tienen dificultades para comprenderla^(23)^.

Se expresó el deseo de obtener más información, lo que indica que los participantes reconocían la necesidad de comprender mejor cómo sus acciones afectaban no solo a sus condiciones de vida y salud, sino también a la salud colectiva y a la calidad de los recursos disponibles en el entorno. Por lo tanto, es fundamental promover iniciativas educativas dialógicas y emancipadoras para capacitar a la población y facilitar su participación, sensibilizándola para crear redes colaborativas con el objetivo de fortalecer la salud y la calidad de vida, en consonancia con la preservación del medio ambiente^(33,34)^.

Las limitaciones de este estudio se refieren al hecho de que se desarrolló en un escenario específico, con características económicas, políticas, socioculturales y territoriales particulares, lo que restringe la amplitud geográfica de los datos. Sin embargo, el conjunto de resultados y reflexiones que se desprenden de él pueden contribuir significativamente a las áreas de enfermería y salud colectiva, al proporcionar subsidios para comparar e interpretar resultados de otras investigaciones en contextos similares, mejorar las acciones de cuidado en el contexto ribereño en relación con la salud ambiental y desarrollar políticas públicas, programas educativos y otras iniciativas dirigidas a la promoción de la salud y la protección ambiental.

## CONCLUSIÓN

Se ha demostrado que la ASA de los ribereños de la Amazonia paraense implica la atribución de significados al entorno que los rodea, la adopción de prácticas para cuidar el medio ambiente y la salud, y el acceso a información sobre el tema. Por lo tanto, es fundamental que los ribereños comprendan cómo sus acciones repercuten en la salud individual y colectiva, involucrando la interrelación entre prácticas de higiene, saneamiento básico y protección ambiental. Sin embargo, factores como el acceso limitado a la información, las condiciones socioeconómicas desfavorables y la falta de iniciativas educativas cualificadas pueden dificultar la ASA, interfiriendo en la capacidad de tomar decisiones ante las condiciones del entorno.

Las repercusiones de la ASA en la salud de los ribereños pueden ser significativas, contribuyendo a prevenir enfermedades y promover un estilo de vida saludable. En esta perspectiva, sensibilizar sobre la necesidad de preservar el medio ambiente puede fortalecer la relación de los ribereños con su entorno, fomentando el sentido de comunidad para establecer responsabilidades compartidas. Por lo tanto, invertir en educación y en la cualificación de las acciones realizadas es esencial para empoderar a estas personas y, así, convertirlas en agentes activos en la preservación del medio ambiente y en la mejora de sus condiciones de vida y salud.

A partir de los datos, se entiende que será posible generar otros conocimientos científicos, obtenidos con solidez, a través de actividades de investigación en enfermería y áreas afines, para fundamentar o fomentar la creación, implementación y efectividad de políticas públicas dirigidas a las necesidades de estas poblaciones. Al estar adaptadas cultural y socialmente, estas políticas pueden contribuir a la equidad en el acceso a la salud y a la preservación ambiental en territorios históricamente vulnerables.

En este contexto, los profesionales de enfermería, sobre todo los enfermeros, podrán actuar de manera que promuevan la salud, con el fin de que las personas y los grupos accedan, comprendan, evalúen y utilicen los conocimientos sobre salud y medio ambiente para tomar decisiones con conciencia crítica, empoderándose en el cuidado de sí mismos y del medio que los rodea, convirtiéndose en agentes de cambio en sus comunidades. Esto pone de manifiesto el papel del enfermero como educador, debido al contexto social de las poblaciones ribereñas, caracterizado por un bajo nivel educativo y un difícil acceso a la información.

## Data Availability

Los datos que respaldan este estudio pueden solicitarse al autor correspondiente.
